# An intravitreal-injectable hydrogel depot doped borneol-decorated dual-drug-coloaded microemulsions for long-lasting retina delivery and synergistic therapy of wAMD

**DOI:** 10.1186/s12951-023-01829-y

**Published:** 2023-03-01

**Authors:** Wenting Su, Congyan Liu, Xi Jiang, Yanli Lv, Qin Chen, Jiachen Shi, Huangqin Zhang, Qiuling Ma, Chang Ge, Fei Kong, Xiaoqi Li, Yuping Liu, Yan Chen, Ding Qu

**Affiliations:** 1 Affiliated Hospital of Integrated Traditional Chinese and Western Medicine, Nanjing University of Chinese Medicine, Nanjing, 210028 China; 2 Jiangsu Province Academy of Traditional Chinese Medicine, 100 Shizi Road, Nanjing, 210028 China; 3 Department of Ophthalmology, Nanjing Drum Tower Hospital, The Affiliated Hospital of Nanjing University Medical School, Nanjing, 210008 China

**Keywords:** Microemulsions-doped hydrogel, Choroidal neovascularization, Retinal pigment epithelium, Wet age-related macular degeneration, Rhein, Baicalein

## Abstract

Sustained retina drug delivery and rational drug combination are considered essential for enhancing the efficacy of therapy for wet age-related macular degeneration (wAMD) due to the conservative structure of the posterior ocular segment and the multi-factorial pathological mechanism. Designing a drug co-delivery system that can simultaneously achieve deep penetration and long-lasting retention in the vitreous is highly desired, yet remains a huge challenge. In this study, we fabricated Bor/RB-M@TRG as an intravitreal-injectable hydrogel depot for deep penetration into the posterior ocular segment and long-lasting distribution in the retinal pigment epithelium (RPE) layer. The Bor/RB-M@TRG consisted of borneol-decorated rhein and baicalein-coloaded microemulsions (Bor/RB-M, the therapy entity) and a temperature-responsive hydrogel matrix (the intravitreal depot). Bor/RB-M exhibited the strongest in vitro anti-angiogenic effects among all the groups studied, which is potentially associated with improved cellular uptake, as well as the synergism of rhein and baicalein, acting via anti-angiogenic and anti-oxidative stress pathways, respectively. Importantly, a single intravitreal (IVT) injection with Bor/RB-M@TRG displayed significant inhibition against the CNV of wAMD model mice, compared to all other groups. Particularly, coumarin-6-labeled Bor/RB-M@TRG (Bor/C6-M@TRG) could not only deeply penetrate into the retina but also stably accumulate in the RPE layer for at least 14 days. Our design integrates the advantages of borneol-decorated microemulsions and hydrogel depots, offering a promising new approach for clinically-translatable retinal drug delivery and synergistic anti-wAMD treatment.

**Supplementary Information:**

The online version contains supplementary material available at 10.1186/s12951-023-01829-y.

## Background

Wet age-related macular degeneration (wAMD), also known as exudative or neovascular AMD, is becoming a leading reason for visual loss in the elderly population worldwide. In contrast to dry AMD, which is the most common form of AMD characterized by drusen between the inner layer of the retinal pigment epithelium (RPE) and Bruch’s membrane, and geographic atrophy, wAMD is characterized by Bruch’s membrane oxidative stress injury-induced choroidal neovascularization (CNV), which can grow into the RPE or photoreceptor layer, leading to exudative, hemorrhagic detachment, and bumpy lesion. Patients with wAMD may experience a sudden loss of vision, metamorphopsia, or central scotoma [1]. Rhein (RH) has shown great potential in a variety of angiogenesis-associated therapies [2, 3], but its efficacy in treating wAMD has yet to be fully validated. Intravitreal (IVT) injection of anti-vascular endothelial growth factor (anti-VEGF) antibodies has been widely used as the first-line strategy against wAMD because of its high anti-angiogenic efficacy [4, 5]. Unfortunately, approximately one-third of wAMD patients are not responsive to IVT injection of anti-VEGF antibodies. The mainstream views hold that multi-pathways drive the pathological progression of wAMD [6], and the mono VEGF-targeted therapy is somehow ineffective at inhibiting the effects of non-VEGF pathways on the development of wAMD.

Oxidative stress, leading to subretinal hypoxia, has been identified as one of the culprits of CNV, which exacerbates angiogenesis around the RPE and activates the inflammatory pathways [7, 8]. Such a mechanism loop connected by oxidative stress and angiogenesis supports the progression of CNV. Baicalein (BCL) has been employed as a potential treatment for retinal inflammatory edema through activating PI3K/AKT pathway, which reduces mitochondrial and intracellular reactive oxygen species (ROS), repairs oxidative stress injuries, and thereby exerts strong antioxidant [9, 10]. Theoretically, combinational RH and BCL are promising to cut off the mechanism loop of CNV pathology from multiple pathways. However, the pharmaceutical application of RH and BCL is limited due to their poor solubility and bioavailability [11, 12], presenting a challenge in delivering these agents to the retina.

The RPE layer, which is located in the inner part of the posterior ocular segment, is a crucial target for anti-CNV agents. However, the conservative physiological structures of the posterior ocular segment, such as the vitreoretinal interface (VRI) and RPE tight junction of (RPE-TJ) [13, 14], present an ongoing obstacle to drug deep penetration. Despite advancements in pharmaceutical technologies, few commercial IVT-injectable formulations achieved an intravitreal half-life of longer than 7 days [15]. An ideal retina drug delivery system for wAMD treatment should exhibit both deep penetration into the RPE and sustained drug release at lesion sites in the retina, which is still a huge challenge for most current formulations. The vitreous-injectable hydrogel system possessing similar composition to the vitreous can offer a solid foundation for long-lasting retention and sustainable drug release [16, 17]. However, such a physicochemical property of hydrogel results in a highly restricted fluidity in the vitreous. To further improve penetration into the RPE layer, a secondary delivery system with deep penetration capability needs to be integrated into the hydrogel system. Our previous studies have demonstrated that small-sized microemulsions possess a distinctive ability to penetrate deep into a variety of tissues [18, 19], such as tumors, the brain–blood barrier, and intestinal walls. In addition, the microemulsions have displayed the outstanding capacity of multicomponent co-loading [18–20], offering promising carriers for the codelivery of RH and BCL. With this in mind, we proposed a strategy that integrates the advantages of hydrogels and microemulsions for long-lasting retina drug delivery.

**Scheme 1:**
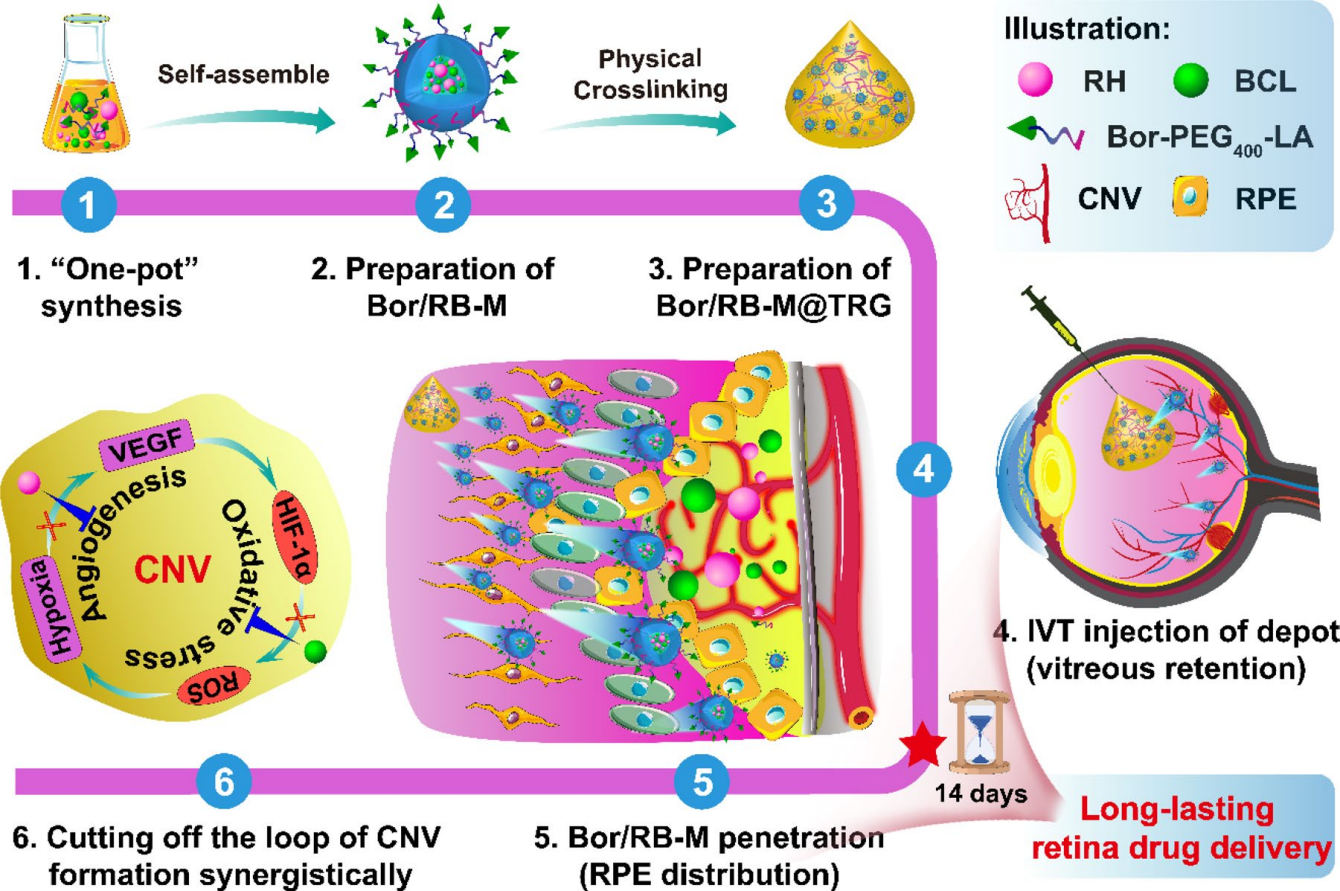
Schematic preparation and sustained retina drug delivery of Bor/RB-M@TRG. Bor/RB-M@TRG composed of Bor/RB-M and a temperature-responsive hydrogel network is prepared by “one-pot” synthesis and physical crosslinking successively. Bor/RB-M@TRG is able to be colonized in the vitreous for at least 14 days and sustainedly release Bor/RB-M to the RPE, thereby synergistically cutting off the CNV formation driven by the “angiogenesis-oxidative stress” loop

Herein, we present Bor/RB-M@TRG, an intravitreal-injectable hydrogel depot doped with borneol-decorated dual-drug-coloaded microemulsions for the continuous and synergistic therapy of wAMD. Bor/RB-M@TRG composed of Bor/RB-M (deep penetration and therapy entity) and temperature-responsive hydrogel network (TRG, the intravitreal depot) is capable of blocking CNV through both anti-angiogenic and anti-oxidative stress pathways. As illustrated in Scheme 1, after a “one-shot” of IVT injection, Bor/RB-M@TRG rapidly transfers to the hydrogel depot in the vitreous and sustainedly releases Bor/RB-M, which can break through the barriers of VRI and RPE-TJ by the inherent deep penetration of the small-sized microemulsion and borneol decoration. Once reached the RPE, RH and BCL co-released from Bor/RB-M could synergistically and continuously hinder the CNV formation driven by the “angiogenesis-oxidative stress” loop. Notably, a single IVT injection of Bor/RB-M@TRG achieved long-lasting accumulation in the RPE for at least 14 days and showed significant anti-wAMD ability among all the groups. This new strategy provides a sustained and efficient drug delivery system with promising potential in retinal disease therapy.

## Materials and methods

### Materials

Rhein (RH) and Baicalin (BCL) were purchased from YuanYe Bio-Technology Co., Ltd (Shanghai, China). Succinic anhydride and PEG_400_ were purchased from Sinopharm Chemical Reagent Co., Ltd, poly(ethylene glycol) monolaurate (PEG_400_-LA) were purchased from YuanYe Bio-Technology Co., Ltd (Shanghai, China). Borneol were purchased from Fujian Green Pine Co., Ltd (Fujian, China). Tert-Butyl hydroperoxide (TBHP) and CoCl_2_ were purchased from Sigma-Aldrich (Shanghai, China). Kolliphor^®^ HS15 and Koliphor^®^ F68 were offered by BASF Co., Ltd (Shanghai, China). Coumarin 6 (C6) as purchased from Aladdin Biochemical Technology Co., Ltd (Beijing, China) Pluronic^®^ F127 was bought from Sigma-Aldrich (Shanghai, China). Labrafil M 1944CS was received as a gift from Gattefossé Co., Ltd (Nanterre Cedex, France). Hyaluronic acid (HA, 10 kDa and 400 kDa) were purchased from Shanghai Yien Chemical Technology Co., Ltd (Shanghai, China). HA (800–1500 kDa and 1500–2500 kDa) were provided by Shanghai Maclin Biochemical Technology Co., Ltd (Shanghai, China). Sudan III was purchased from Sinopharm Group Co., Ltd (Shanghai, China). Ultrapure water was acquired via Elix^®^Essential system (Millipore, Shanghai, China). Other chemicals were used of analytical grade.

### Synthesis of butanediyl borneol (SA-Bor)

Succinic anhydride (SA, 10.0074 g, 0.1 mol) and borneol (Bor, 15.7341 g, 0.102 mmol) were dissolved in 150 mL of anhydrous CH_2_Cl_2_ with 4-dimethylaminopyridine (DMAP, 1.2278 g, 0.01 mol) and stirred for 72 h at room temperature. Subsequently, the solvent was removed by rotary evaporation. The resulting transparent oil was purified by column chromatography (CH_2_Cl_2_/methanol, 70/1, v/v) to yield the transparent crystal SA-Bor, followed by being confirmed by hydrogen spectrum nuclear magnetic resonance (^1^H-NMR) and high-resolution mass spectrometry (HRMS).

### Synthesis of borneol-conjugated polyethylene glycol laurate (Bor-PEG_400_-LA)

SA-Bor (3.1233 g, 3.54 mmol) and PEG_400_-LA (3.0038 g, 4.25 mmol) were dissolved in 50 mL of CH_2_Cl_2_ and stirred magnetically in an ice-water bath. Dicyclohexylcarbodiimide (DCC, 0.7242 g, 3.51 mmol) and DMAP (0.05318 g, 0.44 mmol) were dissolved in 20 mL of CH_2_Cl_2_ and then transferred dropwise to the above resulting mixture, followed by stirring at 0 °C for 1 h and room temperature for an additional 72 h. The reaction mixture was filtered thrice, concentrated, and centrifuged at 8000 rpm to obtain the supernatant. After being precipitated with ice ether at 4 °C for 4 h, Bor-PEG_400_-LA was gained at a yield of 78.4%. The chemical structure was confirmed by ^1^H-NMR (CDCl_3_), HRMS, and Fourier transform-infrared spectroscopy (FT-IR).

### Preparation and characterizations of Bor/RB-M

Three milligrams of RH, 9.42 mg of BCL, 331 mg of Labrafil M 1944CS, 580 mg of HS15, 193 mg of PEG400, and 122 mg of Bor-PEG_400_-LA were stirred strongly at 37 °C overnight. 5 mL of ultrapure water was added dropwise until the solution became homogeneous and transparent with opalescence [21]. Sd III-M and Bor/C6-M were prepared as the similar methods mentioned above, with the exception of replacing the drugs with corresponding probes. The particle size and zeta potential of different microemulsion formulations were measured by dynamic light scattering (DLS, Nano ZS, Malvern, UK). The morphology was evaluated by transmission electron microscopy (TEM, Tecnai 12, Philips, Amsterdam, Netherlands) following previously established methods. Briefly, 15 μL of each sample was deposited on a carbon-coated copper mesh and stained with 1% (v%) phosphotungstic acid. After being dried under infrared light, the sample was observed by TEM. The encapsulation efficiency (EE) and drug loading capacity (LC) of RH and BCL were calculated by the following equations,

$$Encapsulation\, efficiency \left(EE\%\right)=\frac{{W}_{\left(tested\, drug\right)}}{{W}_{\left(feeding \,drug\right)}}\times 100\%;$$

$$ Loading \,capacity \left( {LC\% } \right) = \frac{{W_{\left( {tested \,drug} \right)} }}{{W_{\left( {total \,microemulsion} \right)} }} \times 100\% , $$

where W_tested drug_, W_feeding drug_, and W_total microemulsion_ represent the weight of the tested drug, initial feeding drug, and total microemulsion, respectively. Bor/RB-M were loaded into centrifuge tubes and centrifuged at 13,000 rpm for 10 min to observe any stratification of the microemulsion. Bor/RB-M was placed in PBS at pH 7.4 and the temperature was set at 25 °C. The changes in particle size and PDI of the microemulsions were measured with the DLS on day 1, 3, 5, and 9 to evaluate the stability of the microemulsions. RH and BCL were quantified by high-performance liquid chromatography (HPLC) by the chromatographic conditions reported previously [22].

### Preparation and characterizations of Bor/RB-M@TRG

Five hundred milligrams of F127, 10 mg of F68, 10 mg of HA (1500–2500 kDa), and 2 mL of Bor/RB-M were stirred strongly in an ice-water bath for 4 h and stored in a refrigerator for later use. A similar process was used to prepare Sd III-M@TRG and Bor/C6-M@TRG. The kinetics of dissolution of the gel was observed using a film-free dissolution method as reported previously [23]. Briefly, 1 mL of Sd III-M@TRG solution was injected into 20 mL of phosphate-buffered saline (PBS) at 37 °C, followed by shaking at 100 rpm and acquiring the pictures at the predetermined time intervals. The rheology of Bor/RB-M@TRG and Blank TRG was analyzed by using an Anton Palmer MCR302 rheometer (TA Instruments, Graz, Austria) in the oscillatory mode. The samples were placed on a parallel plate (40 mm diameter) with a gap of 31 mm for measurements. The storage moduli (G′) and loss moduli (G″) were monitored as a function of temperature, at a frequency of 1 Hz and a strain of 1%. The morphology of the hydrogels was studied with scanning electron microscopy (SEM) by using the manipulation protocol.

### Retention in simulated vitreous

A simulated vitreous (SV) was prepared using a previously established method [24]. In brief, a 0.4% w/w agarose solution was made by dissolving agarose in PBS (pH 7.4) and boiling it until completely dissolved. The hot solution was then mixed with 0.5 g of hyaluronic acid (HA) with molecular weight ranging from 1500 to 2500 kDa, and stirred until a homogeneous mixture was obtained. Finally, 30 μL of a 0.02% w/v sodium azide solution was added to the mixture and allowed to cool to room temperature. Sudan III-labeled microemulsion (Sd III-M) and Sd III-M@TRG (based on 800–1500 and 1500–2500 kDa HA) were prepared using Sudan III as the probe. 0.1 mL of Sd III-M and Sd III-M@TRG were added to the simulated vitreous and the tubes were placed in a water bath at 37 °C. The retention of the drugs in the simulated vitreous was observed daily and photographed.

### In vitro drug release

To measure the release rate of Bor/RB-M and Bor/RB-M@TRG, 1 mL of each was placed in a dialysis bag with a molecular weight cut-off of 12,000 Da and immersed in 150 mL of PBS containing 0.5% Tween 80 at 37 °C. At predetermined intervals, 1 mL of the release medium was withdrawn, diluted with 3 times the volume of methanol, and quantified using HPLC. The accumulative release rate was calculated by the following formula, accumulative release (%) = (C_sample_ × f × V)/W_feeding_ × 100%, where C_sample_ represents the concentration of the sample, f represents the dilution factor, V represents the volume of the release medium, and W_feeding_ represents the weight of initial feeding drug.

### Cell culture

The human retinal pigment epithelial (ARPE-19) and human umbilical vein endothelial cells (HUVECs) were cultured with DMEM/F-12 medium and high-sugar DMEM medium, respectively. The culture medium was supplemented with 10% (v%) of fetal bovine serum (FBS) and 1% (wt%) antibiotics (100 U/mL penicillin and 100 mg/mL streptomycin). The cells were cultured in a cell incubator (311, Thermo-Fisher Scientific, MA, USA) at 37 °C in a 5% CO_2_ atmosphere with 90% relative humidity.

### Cytotoxicity assay

ARPE-19 cells (2 × 10^4^ cells/well) were seeded into 96-well plates for 24 h, and then treated with 100 μL of RH, BCL, R + B (a mixture of RH and BCL), RB-M, and Bor/RB-M at a concentration of 0.5 and 1 μM for RH and BCL, respectively. After 6 h of the treatments, H_2_O_2_ (400 μM) was incubated with the cells for an additional 20 h. The medium was replaced with 100 μL DMEM/F-12 and 10 μL MTT solution, which was left to incubate for 4 h. The formed formazan crystals were dissolved in 100 μL of dimethylsulfoxide and the absorbance at 490 nm was recorded by using a microplate reader (Varioskan Flash; Thermo Fisher Scientific, MA, USA). Cell viability (%) was calculated using the following equation,

$$Cell \,viability\left(\%\right)=\frac{{Absorbance}_{ \left(sample\right)}}{{Absorbance}_{ \left(control\right)}}\times 100\%.$$

### Cell apoptosis

ARPE-19 cells (4.2 × 10^5^ cells/well) seeded into 6-well plates were incubated for 24 h and then treated with RH, BCL, R + B, RB-M, and Bor/RB-M at an RH and BCL concentration of 0.5 and 1 μM, respectively. After 6 h of the treatment, the cells were co-incubated with H_2_O_2_ (400 μM) for a further 20 h. After incubation, the cells were rinsed with PBS thrice and trypsinized to the single-cell suspension, followed by staining with an Annexin V-FITC/propidium iodide (PI) detection kit. The stained cells were analyzed immediately using flow cytometry (CytoFLEX, Beckman Coulter, FL, USA) by counting 10,000 events.

### Cellular uptake

ARPE-19 cells (4.2 × 10^5^ cells/well) seeded into 6-well plates were incubated for 24 h. The cells were then exposed to H_2_O_2_ (400 μM) for 20 h and treated with R + B, RB-M, with RH and BCL concentrations of 350 and 700 μM, respectively. After 4 h of incubation, the cells were rinsed with PBS thrice and lysed with SDS (0.5%) at 37 °C. The amount of intracellular drug was quantified with HPLC and cellular protein was determined with a BCA detection kit. Cellular uptake (μg/mg) = A_drug_/A_protein_, where A_drug_ and A_protein_ represent the amount of intracellular drug and cellular protein, respectively. The cells were also treated with C6, C6-M, and Bor/C6-M (286 nM) for 4 h. The intracellular fluorescence images of each well were acquired by a fluorescent inverted microscope (IX71, Olympus, Japan).

### Internalization mechanism studied by confocal laser scanning microscope (CLSM)

ARPE-19 cells (3 × 10^5^ cells/well) seeded into 6-well plates were incubated for 24 h and then treated with H_2_O_2_ (400 μM) for an additional 22 h. After the treatment, the cells were co-incubated with free C6, C6-M, and Bor/C6-M (429 nM) for 2 h. Following incubation, the cells were rinsed with PBS thrice, fixed with 4% paraformaldehyde for 30 min, and then permeabilized with 0.1% Triton X-100 for 5 min. The cells were blocked with 1% BSA, incubated overnight with the rabbit anti-human ZO-1 monoclonal primary antibody (ab221547, Abcam), and stained with the fluorescent secondary antibody Goat Anti-Rabbit IgG (H + L) Fluor 594-conjugated (affinity, S0006, USA). The cells were observed using CLSM (TCS SP8, Germany).

### Scratch assay

HUVECs seeded in a 6-well plate were cultured until 100% coverage and treated with 20 μL of streptomycin (1 mg/mL) for 2 h, followed by scoring with a pipette tip. After treatment with different formulations (RH, BCL, R + B, RB-M, and Bor/RB-M) for 6 h, the cells were cultured for further 24 h, and then the scratch images were acquired before and after the treatments. The wound recovery rate was calculated by the following formula,

$$ Wound \,recovery\left( \% \right) = \frac{{A_{0h} - A_{24h} }}{{A_{0h} }} \times 100\% , $$

where A_0 h_ and A_24 h_ are the scratch area at 0 h and 24 h, respectively. The RH and BCL concentrations were set at 0.5 μM and 1.0 μM for each group, respectively.

### Tube formation assay

HUVECs seeded on matrigel-coated 96-well plates at a density of 3 × 10^4^ cells/well were treated with various formulations (RH, BCL, R + B, RB-M, and Bor/RB-M) for 8 h. The RH and BCL concentrations were set at 0.5 μM and 1.0 μM for each group, respectively. Bright-field images were acquired at the center of each well using an inverted microscope when the endothelial tubes were formed. The degree of tube formation was determined by counting the number of tube-like structures manually. Each experiment was repeated at least three times. The analysis of tube formation was performed using the Angiogenesis Analyzer in the Image J software (NIH, USA).

### Transwell assay

A total of 100 μL of HUVEC suspension containing 5 × 10^4^ cells was seeded into the 8.0-μm wells of the Transwell system (Corning, USA) either with or without matrigel and treated with various formulations. 700 μL of culture medium containing 20% (v%) FBS was added to the lower chamber. After 24 h of the treatment, the cells in the lower chamber were fixed with 4% paraformaldehyde for 10 min and then stained with 1% crystalline violet dye for an additional 10 min. The migration of cells from the upper chamber was observed under a fluorescence inverted microscope (IX71, Olympus, Japan). The migrated cells were counted by using the Image J software (NIH, USA).

### ELISA test

HUVECs or ARPE-19 (2.5 × 10^5^ or 4.2 × 10^5^ cells/well) seeded into 6-well plates were treated with RH, BCL, R + B, RB-M, and Bor/RB-M for 6 h. The RH and BCL concentrations were set at 0.5 μM and 1 μM for each group, respectively. After 6 h of the treatment, the cells were co-incubated with CoCl_2_ (100 μM) or H_2_O_2_ (400 μM) for a further 20 h. The cell supernatants were collected and studied the concentration of VEGF, tumor necrosis factor-α (TNF-α), interleukin-1β (IL-1β), malondialdehyde (MDA), and superoxide dismutase (SOD) by ELISA kits. Each experiment was performed in triplicate.

### Western blot analysis

The treated cells were lysed by adding 50 μL of RIPA lysis buffer to each well and incubating the samples in an ice bath for 30 min. The samples were then centrifuged at 13,000 rmp for 10 min to obtain the cell protein, followed by quantifying using a BCA protein detection kit (Thermo-Fisher, Beijing, China). The samples were mixed with RIPA loading buffer, heated at 100 °C for 10 min, and stored at − 80 °C until western blot analysis. An equal amount of protein from each sample was loaded onto 10% SDS-PAGE gels, transferred to nitrocellulose membranes, and analyzed using an anti-HIF-1α antibody (ab179483, Abcam) according to a standard protocol. The quantification was performed by ImageJ software (NIH, USA).

### ROS and Ca^2+^ determination

ARPE-19 cells were cultured at a density of 4.5 × 10^5^ cells/well in 6-well plates for 24 h, and treated with RH, BCL, R + B, RB-M, and Bor/RB-M for an additional 6 h, with RH and BCL concentrations set at 0.35 μM and 0.7 μM respectively. Afterward, the cells were exposed to either TBHP (200 μM) or CoCl_2_ (200 μM) for 20 h and then stained with DCFH-DA (3 μM, S0033S-1, Beyotime Biotechnology, China) or Fluo-4 AM (1.5 μM, S1060, Beyotime Biotechnology, China) in the dark for 30 min. After washing thoroughly, the cell suspension was assayed by using flow cytometry (CytoFLEX, Beckman Coulter, FL, USA). Each experiment was performed in triplicate.

### Penetration in 3D cell sphere

Five hundred thousand HUVECs were seeded into the agarose-coated 96-well plates for 21 days to form a 3D cell sphere [25]. When reached a diameter of 1 mm, the 3D cell sphere was treated with the free C6, C6-M, and Bor/C6-M at a C6 concentration of 150 ng/mL for 12 h. And then, the cell spheres were washed with PBS thrice, fixed with 4% paraformaldehyde for 30 min, and observed using a CLSM (TCS SP8, Germany) in z-stack mode at a scanning interval of 10 μm.

### Intravitreal retention and penetration in vivo

Eight-week-old male health SD rats weighing 260 ± 20 g were anesthetized with tribromoethanol (1 mg/kg, Sigma-Aldrich, Beijing, China) and then intravitreally injected with the free C6, C6-labeled RB-M (C6-M), C6-labeled Bor/RB-M (Bor/C6-M), and C6-labeled Bor/RB-M@TRG (Bor/C6-M@TRG). Each formulation was diluted with saline to a C6 concentration of 43 nM. A 30-gauge needle was gently inserted at 45° relative to the scleral surface. Subsequently, a 32-gauge needle attached to a 10 μL Nanofil syringe (World Precision Instruments, Sarasota, FL, USA) was then gently inserted along the pinhole to inject various C6 formulations. The eyeballs were removed at the predetermined time intervals (1, 2, 4, 10, and 14 days), and prepared the 12 μm-thick frozen slices for imaging by using CLSM (TCS SP8, Germany).

### 532 nm-laser-induced CNV mice model

The 532 nm-laser-induced CNV mice model was established according to a protocol from Ying Tian and coworkers’ group [26] but with some modifications. Prior to the construction of the CNV model, C57BL/6J mice weighing ~ 20 g were first dilated with tropicamide eye drops thrice and then anesthetized with and then Avertin^®^ at a dose of 20 mL/kg. Next, 4 laser spots surrounding the optic disc were burned with an ophthalmic multiwavelength laser emitter (Lumenis Novus Varia) integrated into a slit lamp (Lumenis 1000). Laser parameters were predetermined as follows, laser interval time: 50 ms; laser duration: 100 ms; laser power: 150 mW; spot size: 50 μm. Once the bubbles appear immediately at the laser sites, it is determined that the modeling is successful.

### Anti-wAMD efficacy in vivo

Twenty CNV model mice were randomly divided into five groups as follows, (1) saline (model); (2) R + B; (3) Bor/RB-M; (4) RB-M@TRG; (5) Bor/RB-M@TRG. At 24 h post laser induction, each mouse received intravitreal administration of 1 μL of the above-mentioned formulations with concentrations of 200 μM of RH and 400 μM of BCL by using an ophthalmic surgical microscope (YZ20P5, 66VT, China). The CNV lesions were monitored on day 14 using optical coherence tomography (OCT). To monitor the leakage of neovascular areas, the mice were intraperitoneally injected with sodium fluorescein, followed by observation using Micron IV to capture the CNV in the fundus. Afterward, the eyeballs of the CNV model mice were collected and fixed with 4% paraformaldehyde (PFA) for 1 h at room temperature. After removal of the anterior segment, the RPE-choroid complex layer was carefully separated from the enucleated mouse eye and cut into four petals, followed by staining with IB4 (15 μg/mL, Sigma-Aldrich, Beijing, China) for 12 h and capturing the fluorescence images by using a multiphoton CLSM (STELLARIS & DIVE, Leica, Germany).

### Pathological section studies

On day 15 post the IVT injection, the eyeballs were harvested from the CNV model mice and embedded with optimal cutting temperature compound to prepare the frozen sections. Continuous slicing at 50 μm intervals between the optic papilla and sclera cornea was performed to visualize the CNV lesions. Hematoxylin and eosin (HE) staining of the eyeball section was performed with the classic protocol. To investigate the apoptosis in the posterior ocular segment resulting from the CNV, the eyeball sections were fixed with 200 μL of 4% paraformaldehyde for 1 h at room temperature and then stained with TdT-mediated dUTP nick end labeling (TUNEL) kits at 4 °C overnight. After being rinsed thrice with PBS, the section was immediately observed with a fluorescent inverted microscope (IX71, Olympus, Japan).

### Biocompatibility studies

Twelve healthy SD rats weighing ~ 250 g were randomly divided into three groups. Each rat received an intravitreal administration of saline, Blank TRG, or Bor/RB-M@TRG, with a concentration of 200 μM RH and 400 μM BCL and a volume of 2 μL. 21 days after the injections, the rats were euthanized under anesthesia, and the eyeballs were collected for section preparation. The HE-staining was performed to assess potential damage to the retina from the carriers or formulations. To evaluate any potential blood toxicity, a hemolysis study was conducted as previously reported [27]. Briefly, Bor/RB-M at different concentrations were incubated with red blood cells of SD rats diluted with saline to 2% (v/v) for 2 h at 37 °C and centrifuged at 2000*g* for 10 min. Saline and pure water were set as the negative and positive control groups, respectively. The supernatant was measured at a wavelength of 540 nm with a microplate reader. The hemolysis rate (%) was calculated using the following equation,

$$Hemolysis \,rate\left(\mathrm{\%}\right)=\frac{{A}_{sample}-{A}_{negative \,control}}{{A}_{positive\, control}-{A}_{negative\, control}}\times 100\mathrm{\%},$$

A_sample_, A_negative control_, and A_positive control_ represent the absorption of the sample, negative control, and positive control, respectively.

### Data statistics

All data are presented as mean ± standard deviations (SD). Statistical analysis among groups was conducted using a one-way analysis of variance (ANOVA). *P < 0.05 and **P < 0.01 were considered significant and extremely significant differences, respectively.

## Results and discussion

### Synthesis and characterizations of Bor-PEG_400_-LA

**Fig. 1:**
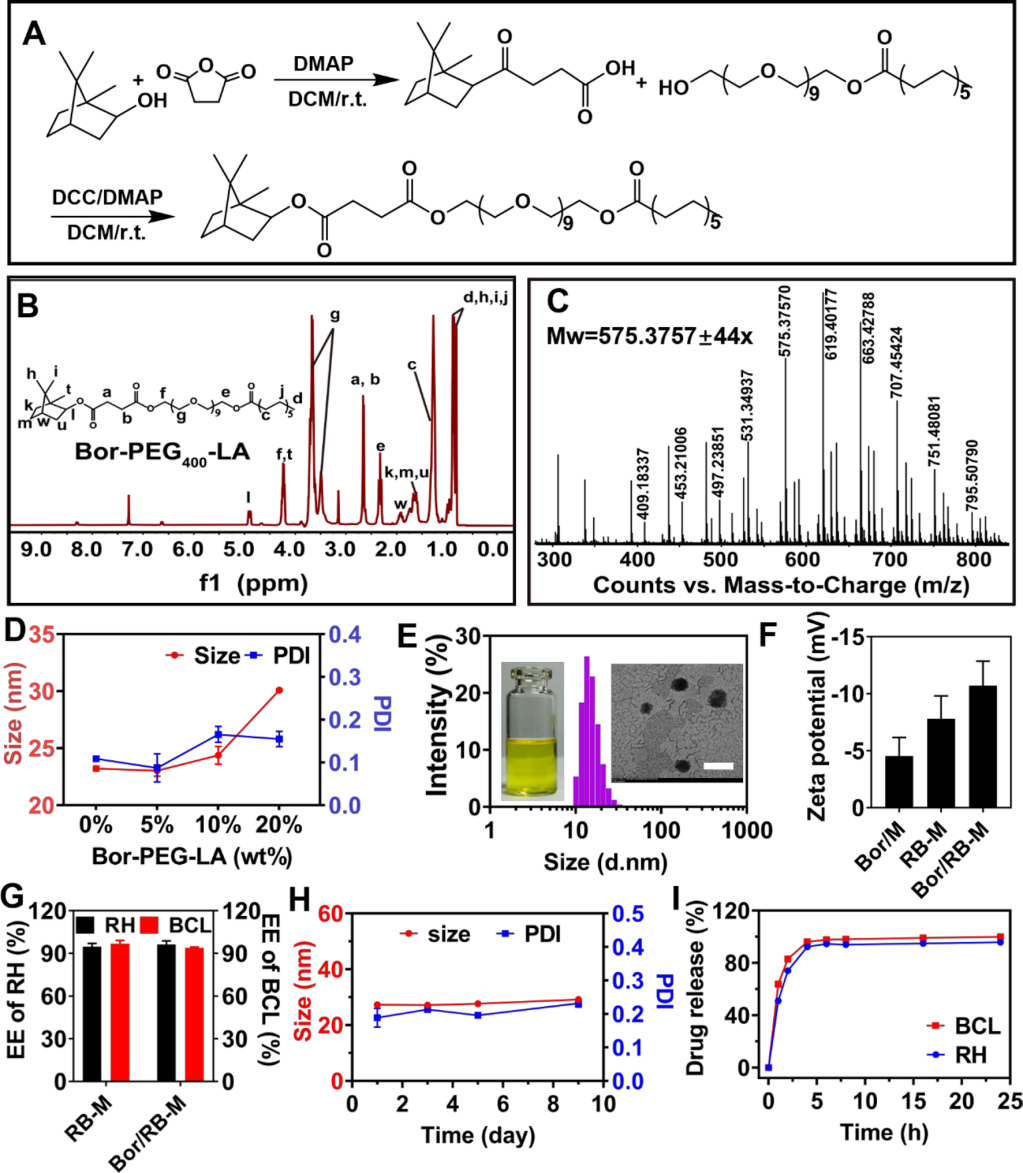
Preparation and characterizations of Bor/RB-M. **A** Synthetic route of Bor-PEG_400_-LA, **B** ^1^H NMR spectrum, and **C** HRMS analysis of Bor-PEG_400_-LA. **D** Particle size and PDI of Bor/RB-M with different feeding of Bor-PEG_400_-LA. **E** Size distribution of Bor/RB-M studied DLS. The left and right inserted pictures are Bor/RB-M solution and its TEM image. The bar is 100 nm **F** Zeta potential of the microemulsions. **G** EE of RH and BCL in RB-M and Bor/RB-M. **H** Stability of Bor/RB-M within 9 days. **I** Release profile of BCL and RH from Bor/RB-M within 24 h. All the data are shown as mean ± SD, n = 6

Borneol (Bor) is a hydrophobic monoterpene small-molecule compound with the proven promotion of tissue deep penetration [28]. A borneol-modified tanshinone IIA liposome was shown to reduce the expression of ICAM-1 and improve the drug bioavailability drug through the blood–brain barrier [29]. In addition, Bor was also able to alter the arrangement of phospholipid molecules in cell membranes and open tight junctions between cells [30–32]. To decorate the microemulsion with borneol, amphiphilic Bor-PEG_400_-LA was synthesized through conjugating PEG_400_-LA and borneol using succinic anhydride as a linker (Fig. 1A). The ^1^H NMR spectrum (Fig. 1B) showed the presence of intense signals in the range of δ (ppm) 3.42 to 3.68, which are attributed to the PEG backbone. The peaks belonging to the aliphatic chain hydrogens of LA were observed at δ (ppm) 0.83 to 0.98. The peaks at δ (ppm) 2.64 were attributed to the linker, while the characteristic chemical shift at δ (ppm) 1.50 to 1.90 and 4.86 was attributed to borneol. The HRMS result presented the expected molecular weight of PEG derivatives at 575.3757 ± 44x (Fig. 1C). Besides, the FT-IR spectrums also showed characteristic absorption peaks corresponding to ester bonds, aliphatic chains, and bridged rings (Additional file 1: Figure S1). These results indicate a successful synthesis of Bor-PEG_400_-LA.

### Preparation and characterizations of Bor/RB-M

The particle size of the microemulsion is crucial for its deep penetration into the retina. Among the excipients, the surfactant is the decisive factor for the particle size of the microemulsion. As shown in Additional file 1: Figure S2, the average size of HS15-based microemulsion was 25.26 ± 0.02 nm, which was significantly smaller than that of RH40-based microemulsions. After incorporating Bor-PEG_400_-LA, the particle size of borneol-decorated blank microemulsions (Bor/M) was comparable to that without borneol modification until the feeding increased up to 10% (wt%, Fig. 1D). Bor/RB-M loading 0.24% (wt%) RH and 0.76% (wt%) BCL appeared as a yellow transparent solution with a narrow particle size distribution (Fig. 1E). Bor/RB-M presented a spherical structure with a particle size of ~ 25 nm, which was similar to that of Bor/M and RB-M (Fig. 1E, Additional file 1: Figures S3, and S4). The zeta potential of Bor/RB-M was − 10.7 ± 2.2 mV, which was significantly lower than that of Bor/M and RB-M (Fig. 1F). The EE of RH and BCL were both beyond 90% in Bor/RB-M and RB-M prepared by the “one-step-microemulsion” method (Fig. 1G). The Bor/RB-M also maintained stability and homogeneity of particle size and PDI at room temperature for 9 days (Fig. 1H). BCL and RH were synchronously released from Bor/RB-M within 6 h (Fig. 1I), which is probably due to their similar physicochemical properties.

### Preparation and characterizations of Bor/RB-M@TRG

**Fig. 2:**
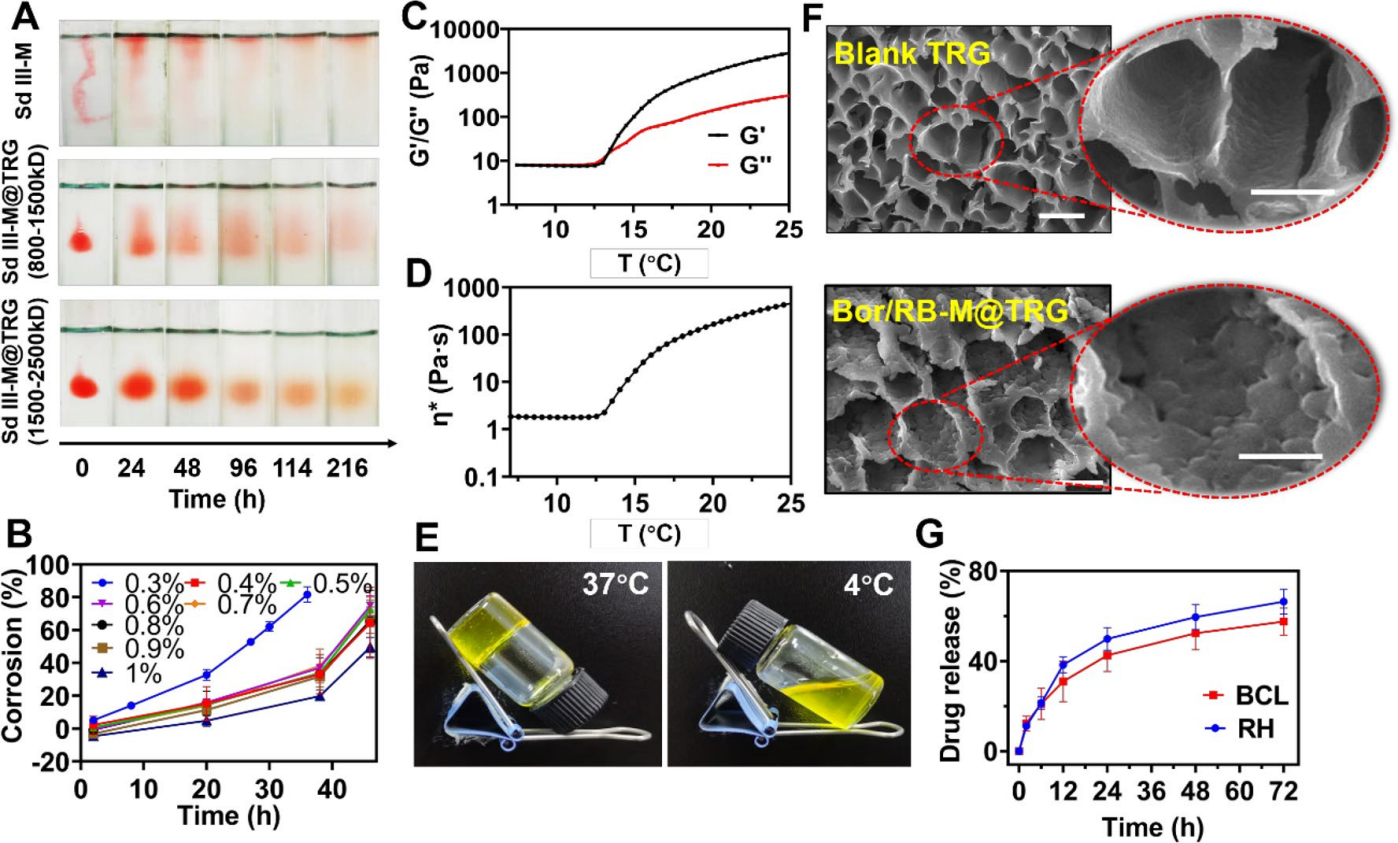
Preparation and characterizations of Bor/RB-M@TRG. **A** Stability of Sd III-M@TRG in the SV. **B** Corrosion studies of Bor/RB-M@TRG prepared with different ratios of HA within 48 h. **C**, **D** Rheological tests of Bor/RB-M@TRG. **E** Solution-gel transition of Bor/RB-M@TRG at 37 °C and 4 °C, respectively. **F** SEM images of blank TRG and Bor/RB-M@TRG. The bar is 20 μm. **G** Release profile of BCL and RH from Bor/RB-M@TRG within 72 h. All the data are shown as mean ± SD, n = 6

The introduction of HA can improve the rigidity of F127/F68-based temperature-responsive hydrogel (TRG), which is positively correlated with the molecular weight (MW) of HA. As shown in Fig. 2A and Additional file 1: Figure S5, the volume of Sd III-M@TRG incorporated the HA with an MW of 1500–2500 kDa showed no significant dissolution or swelling in the SV in 9 days. With a decrease in the MW of HA, the diffusion was found to accelerate at the observed time intervals, causing Sudan III-M to disperse rapidly without protection from the hydrogel. The weight ratio of HA is also of great importance to corrosion in an aqueous environment. As exhibited in Fig. 2B, incorporating 1% (wt%) HA resulted in the lowest corrosion of Bor/RB-M@TRG within 46 h among all the groups. The rheological results (Fig. 2C, D, and Additional file 1: Figure S6) demonstrated that the phase-transition temperatures of Bor/RB-M@TRG and Blank TRG were approximately 14 °C and 19 °C, respectively, indicating that the excipients of the microemulsion could downregulate the phase-transition temperature of Bor/RB-M@TRG. An ice bath before the IVT injection will be required. Bor/RB-M@TRG switched between the gel and solution state at 37 °C and 4 °C, respectively (Fig. 2E). According to the SEM images exhibited in Fig. 2F, a three-dimensional porous structure was observed in the blank TRG, which provides the storage space for the payloads. After encapsulating Bor/RB-M, the inner wall of the porous structure became rough, inferring that Bor/RB-M was loaded by the hydrogel. Bor/RB-M@TRG released approximately 60% BCL and RH in PBS within 72 h, which is significantly slower than Bor/RB-M (Fig. 2G), suggesting that a sustained release resulted from the protection by the hydrogel. Besides, there was no significant difference between the two drugs in the release profile, indicating a potential codelivery of BCL and RH.

### Repair of RPE damage and cellular uptake

**Fig. 3:**
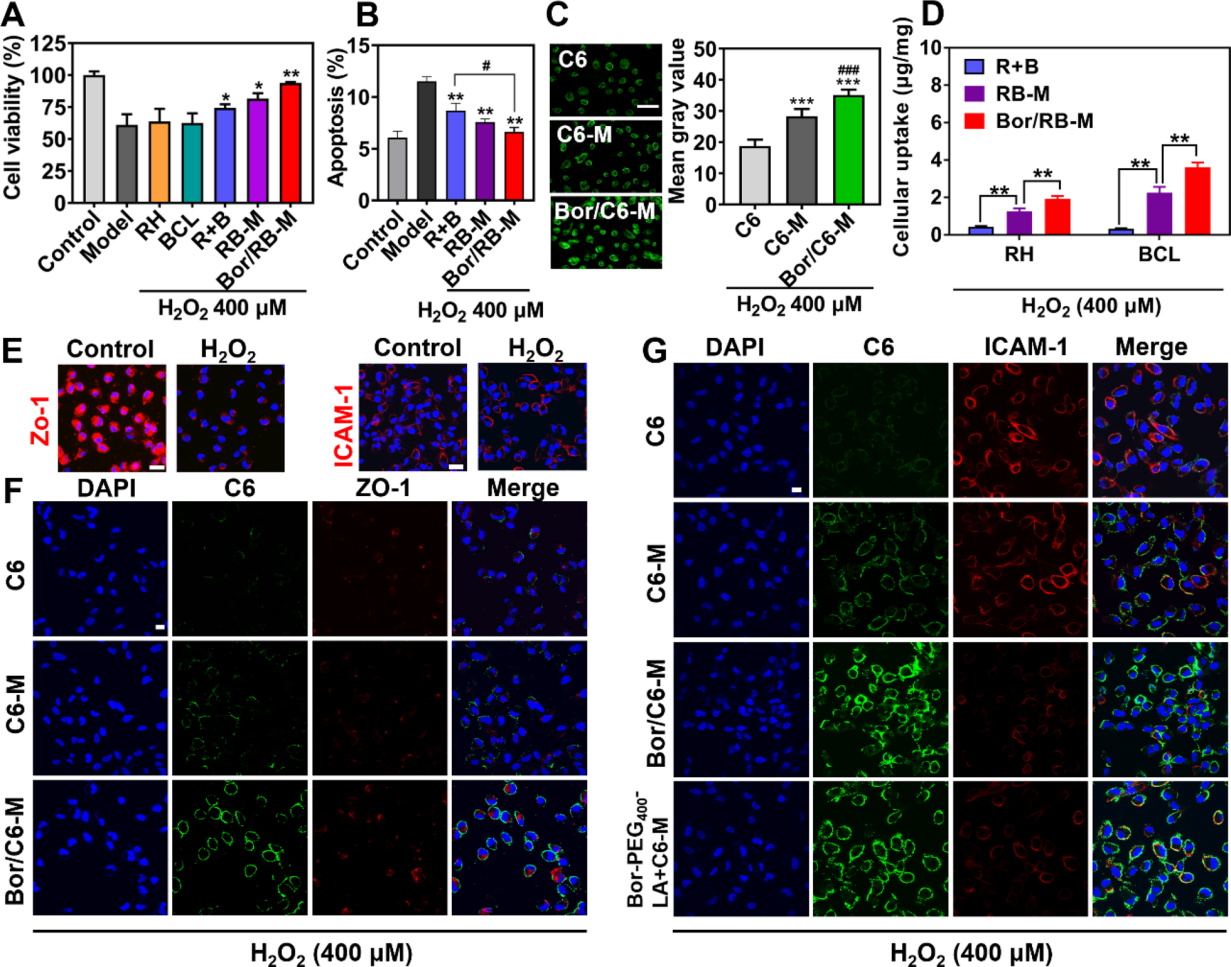
Cellular protection and internalization. **A** Cell viability and **B** cell apoptosis of H_2_O_2_-induced RPE after treatments with different formulations. Data are shown as mean ± SD, n = 6. *P < 0.05, **P < 0.01 vs Model; ^#^*P* < 0.05. Intracellular **C** C6 fluorescence and **D** RH&BCL after H_2_O_2_-induced RPE were treated with different formulations for 4 h. The bar is 100 μm. Data are shown as mean ± SD, n = 6. ***P < 0.001 vs C6; ^###^*P* < 0.001 vs C6-M. **E** Immunofluorescence staining of ZO-1 and ICAM-1 for untreated and H_2_O_2_-induced RPE. The bar is 40 μm. **F** CLSM images of H_2_O_2_-induced RPE treated with different C6-related formulations for 2 h. ZO-1 and formulations were labeled with red and green, respectively. The bar is 20 μm. **G** CLSM images of H_2_O_2_-induced RPE treated with different C6-related formulations for 2 h. ICAM-1 and formulations were labeled with red and green, respectively. The bar is 20 μm

The damage to the RPE caused by oxidative stress is a potential mechanism of wAMD. In comparison with the model, Bor/RB-M significantly improved the viability of H_2_O_2_-induced RPE (Fig. 3A), which is comparable to the untreated cells. In addition, both R + B and RB-M showed synergistic protection toward H_2_O_2_-induced RPE damages compared to the mono RH or BCL group (Fig. 3A). Bor/RB-M remarkably reduced cell apoptosis resulting from the treatment with H_2_O_2_ (Fig. 3B). Likewise, combinational RH and BCL could also cooperatively decrease the H_2_O_2_-induced RPE apoptosis rate (Additional file 1: Figure S7). The intracellular fluorescence of Bor/C6-M-treated cells was 1.24 times and 1.79 times that of the C6-M and free C6 groups, respectively (Fig. 3C). To further assess the cellular uptake of the microemulsions, the quantification of intracellular RH and BCL was also investigated. The cellular uptake of Bor/RB-M reached 3.7 ± 0.2 μg RH/mg and 7.3 ± 0.5 μg BCL/mg, respectively (Fig. 3D), which is significantly higher than that of RB-M. However, the intracellular drug in the R + B-treated cells was hardly detected, suggesting the necessity of microemulsion encapsulation. As shown in Fig. 3E and F, the expression of tight junction protein ZO-1 in H_2_O_2_-induced RPE was significantly lower than in untreated RPE; however, but this decrease did not result in an increased cellular uptake of the free C6 or C6-M. In contrast, Bor/C6-M displayed the highest internalization, suggesting that the cellular mechanism of Bor/C6-M is in a ZO-1-independent manner. Unlike the ZO-1, the expression of ICAM-1 was not influenced after the induction with H_2_O_2_ (Fig. 3E). As shown in Fig. 3G, the strongest intracellular fluorescence in the Bor/C6-M group was observed with a significant decrease in the expression of ICAM-1. Treatment with a mixture of C6-M and Bor-PEG_400_-LA resulted in a significant decrease in ICAM-1 expression and an increased cellular uptake, similar to that of Bor/C6-M. It suggests that a higher internalization of Bor/C6-M is correlated with a lower expression of ICAM-1.

### Anti-angiogenic effects in vitro

**Fig. 4:**
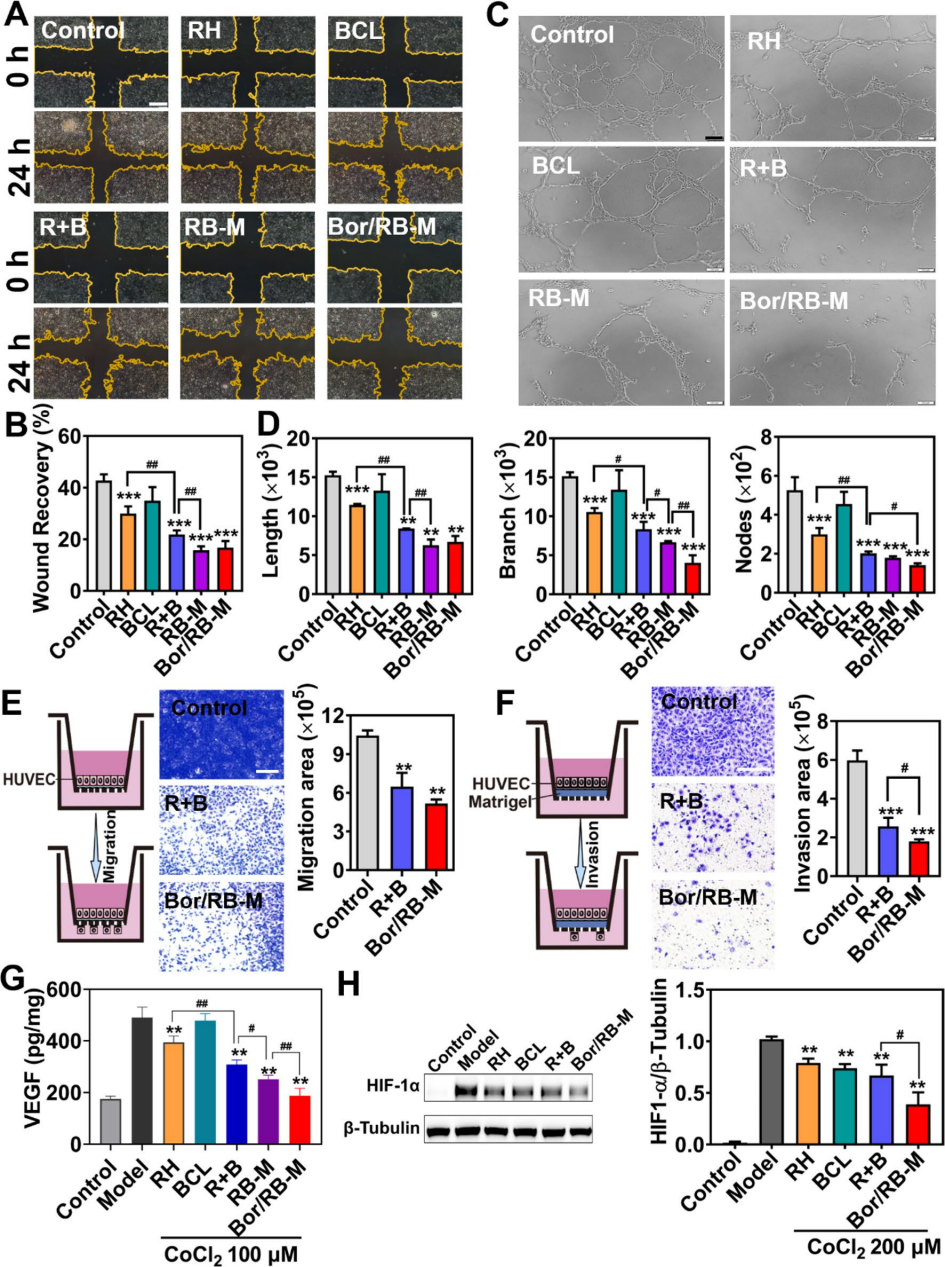
Anti-angiogenic studies in vitro. **A** Scratch tests of the formulations on HUVECs for 24 h and **B** corresponding wound recovery. The bar is 400 μm. **C** Tubule formation after HUVECs were treated with different formulations, and **D** corresponding total length, branch length, and the number of nodes. The bar is 100 μm. Data are shown as mean ± SD, n = 6. **P < 0.01, ***P < 0.001 vs Control; ^#^*P* < 0.05, ^##^*P* < 0.01. **E** Investigations of migration and **F** invasion studied by Transwell. The bar is 100 μm. Data are shown as mean ± SD, n = 6. **P < 0.01, ***P < 0.001 vs Control; ^#^*P* < 0.05. **G** ELISA tests of VEGF secreted by CoCl_2_-induced HUVECs treated with different formulations for 24 h. **H** Western blot analysis of HIF-1α expression of CoCl_2_-induced RPE. Data are shown as mean ± SD, n = 6. **P < 0.01 vs Model; ^#^*P* < 0.05, ^##^*P* < 0.01

As shown in Fig. 4A and B, combinational RH and BCL displayed stronger inhibition of HUVECs migration than either mono RH or BCL. Both Bor/RB-M and RB-M demonstrated a further reduction in wound healing rate compared to the R + B group. It was observed that BCL alone was unable to prevent cell migration, indicating that RH is identified as the major contributor in Bor/RB-M. Bor/RB-M exhibited a significant inhibition in the tube-forming tests among all the groups (Fig. 4C). The typical cord-like and tubular structures with multiple branches were disrupted after the treatment with Bor/RB-M. Although the R + B still presented synergistic inhibition of tubule formation, the microemulsion encapsulation further enhanced such a trend. As for the quantitative assays (Fig. 4D), Bor/RB-M significantly reduced both the branch length and number of nodes of the tubes compared to RB-M. The obtained results verified the significance of combinational strategy, microemulsion encapsulation, and borneol decoration. Besides, the anti-angiogenic effect was validated using a transwell co-culture system. As presented in Fig. 4E, the vertical migration of HUVECs in the R + B and Bor/RB-M group was remarkably suppressed compared to the control group, which is significantly stronger than the mono RH or BCL group (Additional file 1: Figure S8). Likewise, Bor/RB-M notably blocked the invasion of HUVCEs, which is notably stronger than the R + B and RB-M groups (Fig. 4F and Additional file 1: Figure S9). As shown in Fig. 4G, the VEGF secreted by CoCl_2_-pretreated HUVECs was significantly higher than untreated HUVECs. Both R + B and RB-M downregulated VEGF level compared to the mono RH or BCL group. However, Bor/RB-M demonstrated the top performance in decreasing VEGF among all the groups. Furthermore, HIF-1α expressed by CoCl_2_-pretreated RPE was significantly reduced after the treatment with Bor/RB-M compared to the R + B group (Fig. 4H).

### Anti-oxidative stress in vitro

**Fig. 5:**
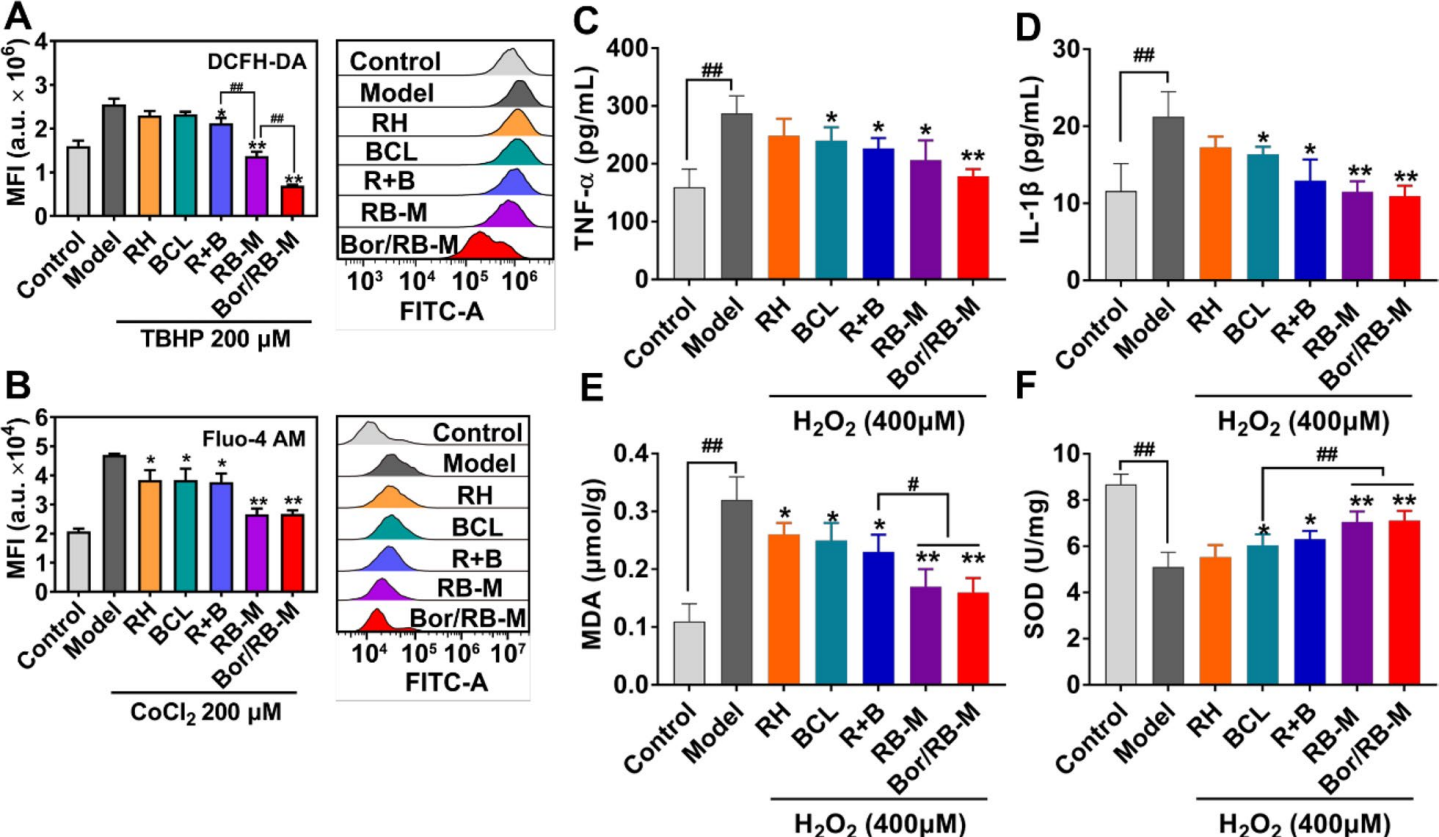
Anti-oxidative stress studies in vitro. **A** Intracellular fluorescence of DCFH-DA (ROS) after TBHP-induced RPE was treated with different formulations. **B** Intracellular fluorescence of Fluo-4 AM (Ca^2+^) after CoCl_2_-induced RPE was treated with different formulations. Data are shown as mean ± SD, n = 4. *P < 0.05, **P < 0.01 vs Model; ^##^*P* < 0.01. ELISA tests of **C** TNF-α, **D** IL-1β, **E** MDA, and **F** SOD secreted by H_2_O_2_-induced RPE treated with different formulations for 24 h. Data are shown as mean ± SD, n = 6. *P < 0.05, **P < 0.01 vs Model; ^#^*P* < 0.05, ^##^*P* < 0.01

As described in the introduction, reducing oxidative stress damage around the RPE is beneficial to block the loop of the “angiogenesis-oxidative stress” pathway. In this part, we investigated the anti-oxidative stress effects of various formulations to evaluate their individual contributions to anti-CNV. As shown in Fig. 5A, the model (cells induced by TBHP only) indeed had a higher ROS level than the control. Combinational RH and BCL displayed stronger inhibition of ROS production compared to the control; however, the two individual compounds failed. Both RB-M and Bor/RB-M further decreased the level of ROS in comparison to the R + B group, which is likely due to the improvement of cellular uptake. Oxidative stress can lead to abnormal cellular energy metabolism and aggregation of disease-related proteins, thereby affecting calcium homeostasis, and triggering extracellular calcium influx and calcium release from the endoplasmic reticulum [33]. As revealed in Fig. 5B, higher intracellular Ca^2+^ was detected in the model (cells induced by CoCl_2_ only) than in the control. BCL alone was able to lower the intracellular Fluo-4 AM signal, and this effect was amplified by the R + B group. As expected, the two microemulsions further decrease intracellular Ca^2+^ concentration on the basis of the R + B group. According to the cytokine tests (Fig. 5C–E), the treatment with BCL was able to significantly reduce the secretion of TNF-α, IL-1β, and MDA by H_2_O_2_-induced oxidative stress model cells, while RH seemed to be ineffective to these cytokines. Both RB-M and Bor/RB-M could further lower the level of MDA compared to the R + B group. Likewise, BCL promoted the secretion of SOD, and the two microemulsions further increase the level (Fig. 5F). All these results from Figs. 4 and 5 suggest that BCL works in cooperation with RH to resist CNV formation, with BCL primarily reducing oxidative stress and RH inhibiting angiogenesis. The improved cellular uptake resulting from microemulsion encapsulation and borneol modification are also the two keys to effective anti-oxidative stress.

### Penetration and retention

**Fig. 6:**
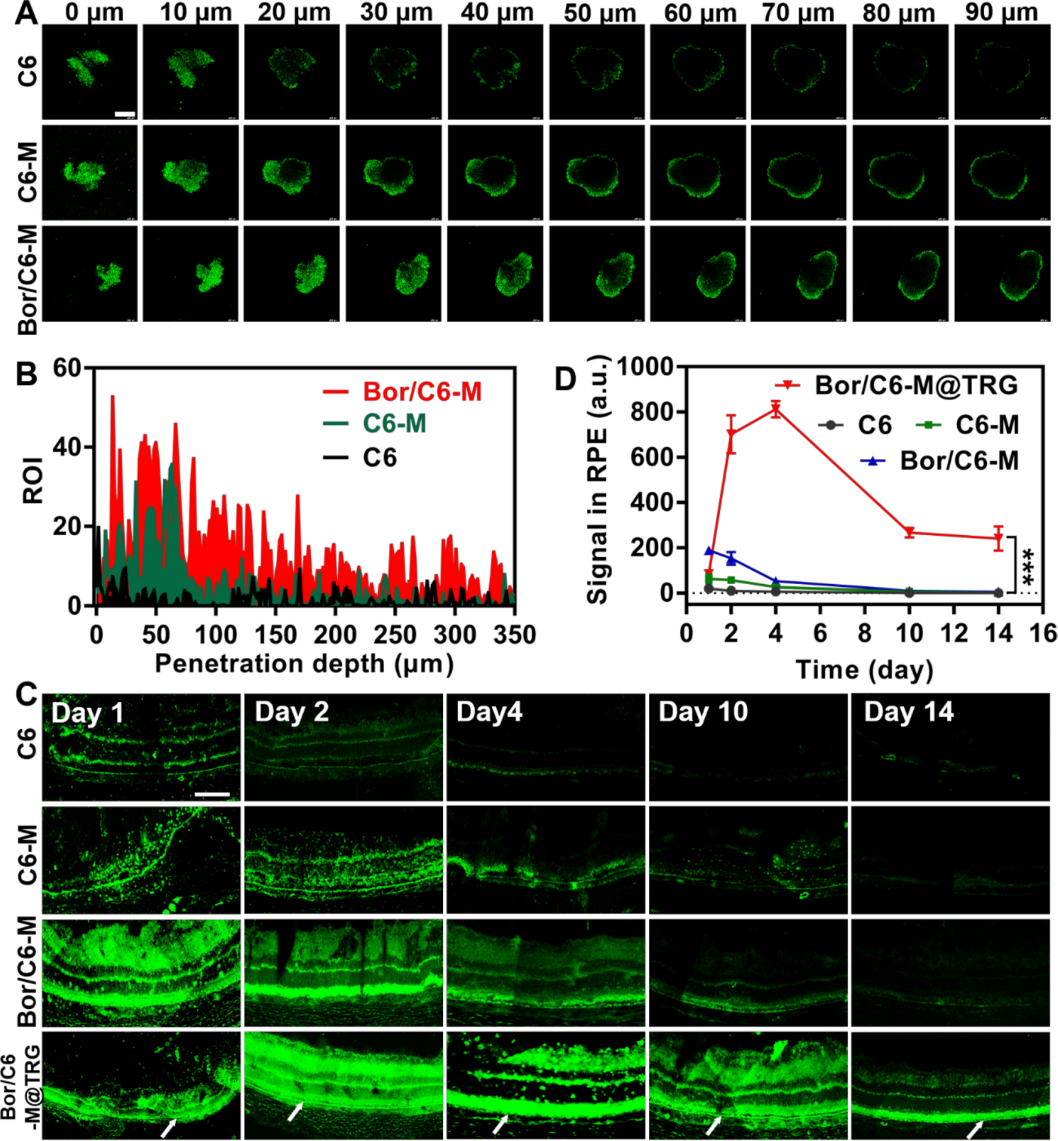
Penetration and retention. **A** CLSM images of 3D HUVECs cell spheres treated with different C6-labeled formulations. The cell spheres at different depths ranging from 0–90 μm were scanned using the z-stack tool. The bar is 400 μm. **B** Corresponding quantification of green fluorescence at the depths ranging from 0–350 μm calculated by ImageJ software. **C** CLSM images of posterior ocular segment sections of SD rats treated with different C6-labeled formulations within 14 days. The white arrows point to the RPE layer. The bar is 400 μm. **D** Corresponding quantification of green fluorescence in the RPE layer. The quantitative area is selected randomly from the three RPE sites, n = 3. ***P < 0.001

Deep penetration of Bor/RB-M and prolonged retention of Bor/RB-M@TRG inside the vitreous are two key factors for sustained drug delivery to the retina. To evaluate the in vitro penetration of Bor-modified microemulsions, the HUVECs 3D cell sphere was established. As shown in Fig. 6A, Bor/C6-M had a significantly deeper penetration depth of ~ 40 μm compared to C6-M, which only had a depth of ~ 10 μm. The semi-quantitative results once again intuitively demonstrated the advantage of Bor/C6-M over other groups in 3D cell sphere penetration (Fig. 6B). To investigate intravitreal penetration and retention and retina distribution, a single IVT injection of various C6-labeled formulations was performed. As shown in Fig. 6C, the free C6 had weak fluorescence in the RPE layer, which only lasted for 1 day. After the treatment with C6-M, a stronger green fluorescence was observed at both the posterior ocular segment and RPE layer at 2 days post IVT injection, while Bor/C6-M further enhanced the green signal at the desired sites. However, the retention time of Bor/C6-M at the RPE was still less than 4 days. Notably, Bor/C6-M@TRG showed overwhelming fluorescence at the posterior ocular segment of the eye from day 2 to day 10, and even fluorescence accumulation in the RPE layer was observed on day 14 (Fig. 6C and D). These results demonstrate the efficiency of the retinal drug delivery and prolonged retention by Bor/RB-M@TRG, creating positive conditions for the synergistic treatment of AMD.

### Anti-wAMD efficacy in vivo

**Fig. 7:**
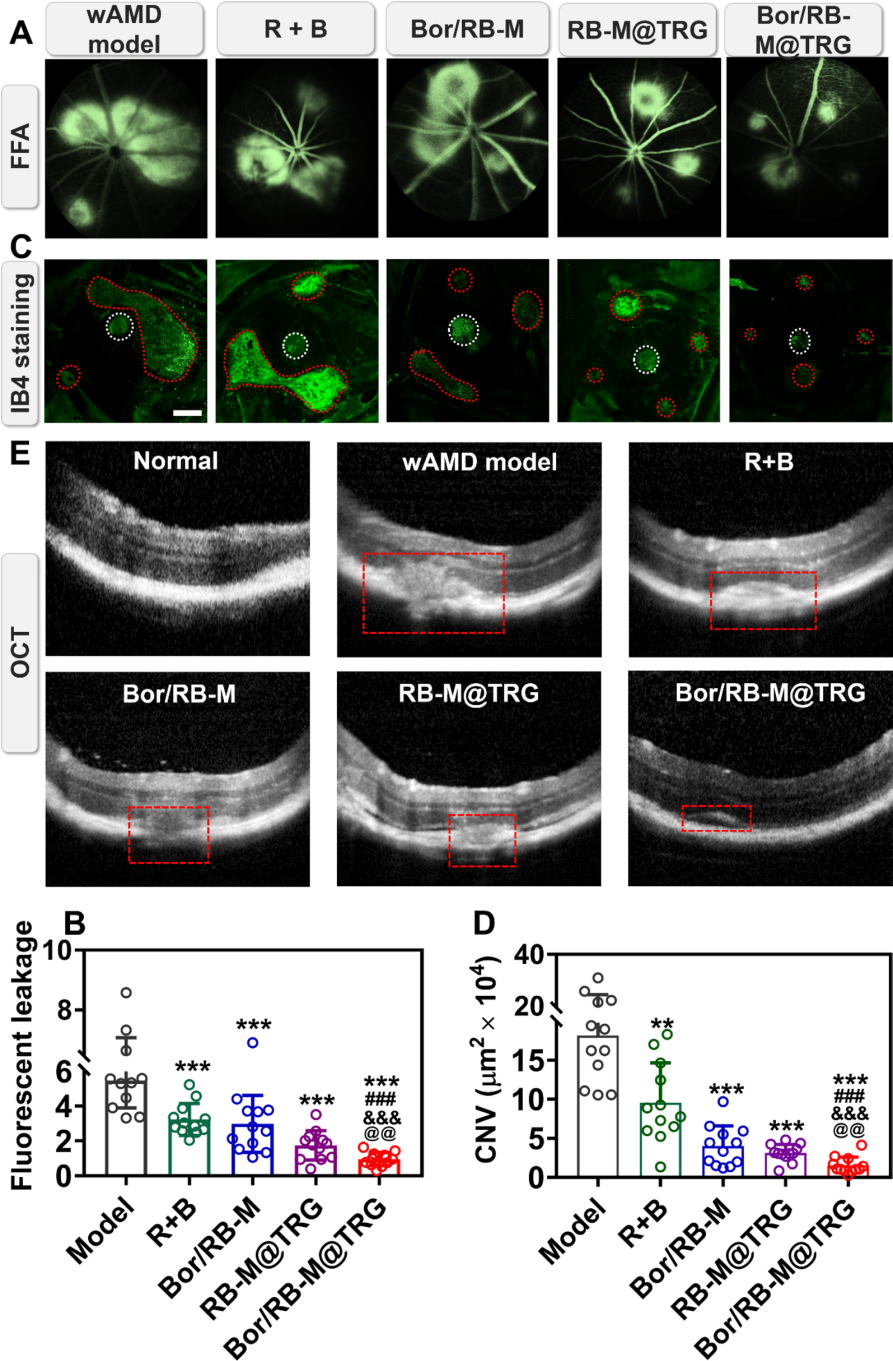
Anti-wAMD efficacy in vivo. **A** FFA images of wAMD model mice treated with different formulations at day 14 and **B** quantitative fluorescence leakage area for each spot calculated by ImageJ. Data are shown as mean ± SD, n = 12. ***P < 0.001 vs Model; ^###^*P* < 0.001 vs R + B; ^&&&^*P* < 0.001 vs Bor/RB-M; ^@@^*P* < 0.01 vs RB-M@TRG. **C** IB4 staining of the RPE-choroid complex layer. The part circled with red is the CNV area, and the part circled with white is the optic disc. The bar is 200 μm. **D** Quantitative CNV area calculated by ImageJ. Data are shown as mean ± SD, n = 12. **P < 0.01, ***P < 0.001 vs Model; ^###^*P* < 0.001 vs R + B; ^&&&^*P* < 0.001 vs Bor/RB-M; ^@@^*P* < 0.01 vs RB-M@TRG. **E** OCT images of wAMD model mice treated with different formulations at day 14. The part circled in red delineates the rupture and bulge in the subretinal space

The aim of this study was to evaluate the effectiveness of Bor/RB-M@TRG in anti-wAMD therapy. In vitro experiments showed that the Bor/RB-M@TRG system had deep penetration toward the RPE layer and prolonged retention in the vitreous, making it a promising candidate for anti-angiogenesis treatment. In this part, in vivo experiments were performed to verify the potential of Bor/RB-M@TRG in treating the wAMD mouse model. As shown in Fig. 7A and B, three laser-induced spots were fused to a huge vascular leak site in the wAMD model group at 14 days post-modeling. To investigate the synergism of RH and BCL in the anti-wAMD therapy in vivo, the mono treatments with RH or BCL were also performed. As a result, neither RH nor BCL displayed sufficient effectiveness in inhibiting the CNV (Additional file 1: Figure S10). In contrast, only two big fluorescence spots were observed in both the R + B and Bor/RB-M group, and the average fluorescence leakage area was noticeably smaller than the wAMD model, indicating promising potential in anti-wAMD treatment. Most importantly, a single IVT injection of Bor/RB-M@TRG remarkably shrunk the four spots compared with the Bor/RB-M, suggesting the significance of prolonged retention in the vitreous. To further verify the importance of deep penetration, the anti-wAMD efficacy of RB-M@TRG was also performed. According to the fundus fluorescence angiography (FFA) images and the quantitative results, the fluorescence leakage in the Bor/RB-M@TRG group was indeed significantly smaller than in the RB-M@TRG. These results demonstrate that the long-lasting retina drug delivery and rational drug combination are of great importance to synergistic therapy of anti-wAMD. Next, the inhibition against the CNV formation in vivo from various formulations was investigated by using the IB4 staining of the RPE-choroid complex layer. As displayed in Fig. 7C and D, the IB4-positive area in the four treated groups was evidently smaller than the wAMD model, which is in accordance with the FFA results. The CNV area after the IVT injection with Bor/RB-M and RB-M@TRG group was further reduced compared with the R + B group, which is ascribed to the deep penetration and prolonged retention, respectively. By comparison, Bor/RB-M@TRG displayed more potent suppression of the CNV formation than other treatments. We next investigated the effects of various formulations on retinal and choroidal lesions by using OCT imaging. As shown in Fig. 7E, an obvious rupture and bulge in the subretinal space were observed from the untreated group, and marginal mitigation was received after the treatments with R + B and Bor/RB-M. In contrast, the CNV pathological features at the RPE and choroid layer were notably relieved in the RB-M@TRG group. Encouragingly, Bor/RB-M@TRG exhibited the strongest repaired ability to the damages of the RPE-choroid layer with a tiny bump. In this study, antioxidant stress therapy acts as a supplement to the combination therapy, although BCL alone cannot significantly reduce VEGF. However, in the real world, the choice between anti-angiogenesis and antioxidant stress therapy should be determined with caution, based on the specific pathological process of the patients. Anti-angiogenesis therapy can rapidly reduce the density of neovascularization, while sustained delivery of antioxidant stress drugs to the retina may reduce the risk of CNV recurrence. The dual drug delivery system designed in this study aims to balance the release of both drugs in an ideal CNV model, minimizing the priority between anti-angiogenesis and antioxidant stress. The effectiveness of the treatments may not be consistent among patients in the real world.

**Fig. 8:**
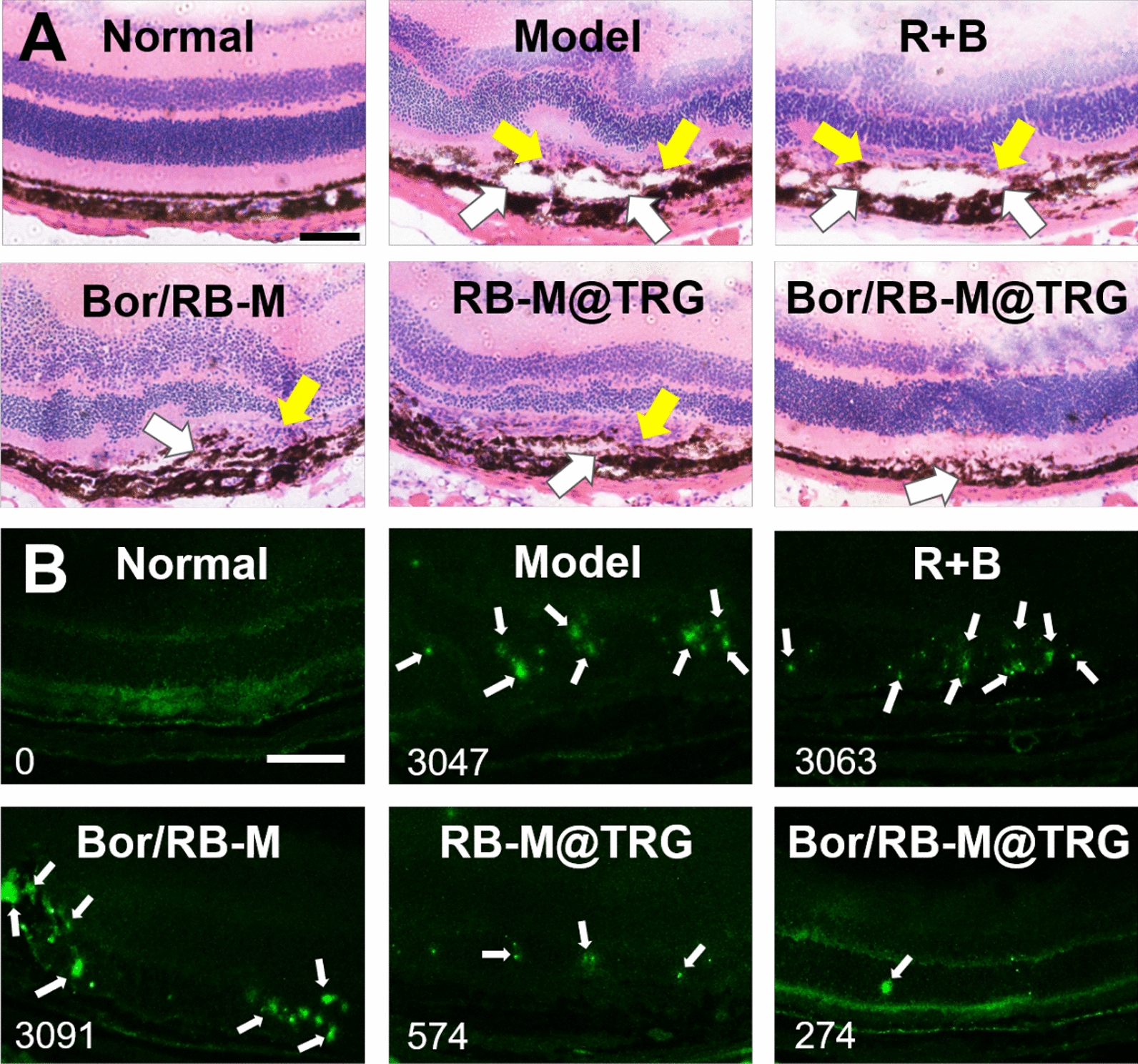
**A** HE staining of eyeballs of mice treated with different formulations at day 14. The white and yellow arrows represent injured RPE and inflammatory cell infiltration. The bar is 200 μm. **B** TUNEL immunofluorescence staining of eyeballs of mice treated with different formulations at day 14. The bar is 200 μm. The apoptosis is primarily assessed based on the presence of significant green fluorescence accumulation points, which are labeled with white arrows. The fluorescence intensity is quantified by Image J and indicated in the lower left corner of each figure

Next, the HE-stained sections of the eyeballs were also employed to assess the impact of various treatments on inhibiting the CNV formation and alleviating inflammatory response. As shown in Fig. 8A, the HE-stained sections from both the model and R + B group presented that the intact structure of the RPE layer was damaged with massive inflammatory cell infiltration. In comparison, Bor/RB-M and RB-M@TRG significantly mitigated the bump between the RPE and choroid and repaired the integrity of the RPE to some extent. The most remarkable results were observed in the Bor/RB-M@TRG group, where the morphology of the RPE layer was similar to that of the normal group, with little infiltration of inflammatory cells. It is well established that oxidative stress and neovascularization caused by CNV can lead to cell apoptosis around the retina and subsequently aggravate CNV formation [34–36]. According to Fig. 8B, the model displayed a significant level of apoptosis in comparison with the normal group. However, Bor/RB-M@TRG exhibited the lowest fluorescence intensity among all the groups, indicating that the cycle of “angiogenesis-oxidative stress” was effectively cut off. Besides, 21-day eye safety was evaluated after a single IVT injection with various formulations. As exhibited in Additional file 1: Figure S11, the HE staining of the eyeballs of healthy SD rats had no pathological changes. Microemulsion system containing lots of surfactants has the risk of hemolysis; however, Bor/RB-M passed the hemolysis tests (Additional file 1: Figure S12), suggesting its acceptable biocompatibility. These results highlight that the combined effects of rational drug combination, deep penetration, and prolonged retention make Bor/RB-M@TRG a promising candidate for treating wAMD.

## Conclusion

Bor/RB-M@TRG was fabricated as an intravitreal hydrogel depot for long-lasting retina drug delivery and boosted therapy of wAMD. Bor/RB-M@TRG achieved deep penetration in the posterior ocular segment and high accumulation in the RPE layer for at least 14 days. A single IVT injection of Bor/RB-M@TRG demonstrated superior inhibition against the CNV in a wAMD mouse model compared to other treatment groups. Rational drug combination and microemulsion-doped intravitreal hydrogel depot are both major contributors to the boosted anti-wAMD efficacy. Such a strategy offers a template tool for long-lasting retinal drug delivery and therapy of retina-related diseases.

## Supplementary Information

**Additional file 1: Figure S1.** FT-IR analysis of Bor-PEG_400_-LA. **Figure S2.** Particle size and PDI of Blank-M with different surfactant. All the quantification data are shown as mean ± SD, n = 3. ***P < 0.001 vs (RH40) Blank-M. **Figure S3.** Size distribution of Bor/M studied DLS. The left and right inserted pictures are Blank-M solution and its TEM image. The bar is 100 nm. **Figure S4.** Size distribution of RB-M studied DLS. The left and right inserted pictures are RB-M solution and its TEM image. The bar is 100 nm. **Figure S5.** Stability of Sd III-M@TRG in simulated vitreous. (A–E) Feedings of HA (800–1500 kDa) were 0.4%, 0.6%, 0.8%, 1.0%, and 1.2%, respectively. (F–J) Feedings of HA (1500–2500 kDa) were 0.4%, 0.6%, 0.8%, 1.0%, and 1.2%, respectively. **Figure S6.** Rheological tests of Blank TRG. (A) Variation curves of G″ and G' with temperature; (B) Variation curve of compound viscosity coefficient and temperature. **Figure S7.** Cell apoptosis of H_2_O_2_-induced RPE after treatments with different formulations. All the quantification data are shown as mean ± SD, n = 6. **P < 0.01 vs Model. **Figure S8.** Migration of HUVECs studied by Transwell. All the quantification data are shown as mean ± SD, n = 6. **P < 0.01 vs Control; ^#^P < 0.05. **Figure S9.** Invasion of HUVECs studied by Transwell. Data are shown as mean ± SD, n = 6. *P < 0.05, **P < 0.01 vs Control; ^##^P < 0.01. **Figure S10.** FFA images of wAMD model mice treated with RH and BCL at day 14. IB4 staining of the RPE-choroid complex layer. The part circled in red is the CNV area, and the part circled in white is the optic disc. The bar is 200 μm. **Figure S11.** HE staining of eyeballs of healthy SD rats treated with different formulations at day 21. The bar is 200 μm. **Figure S12.** Hemolysis tests of Bor/RB-M. Data are shown as mean ± SD, n = 3.

## Data Availability

All data presented in this paper are included in the main text and the additional information.

## Abbreviations

wAMD: Wet age-related macular degeneration
RPE: Retinal pigment epithelium
CNV: Choroidal neovascularization
RH: Rhein
BCL: Baicalein
ROS: Reactive oxygen species
VEGF: Vascular endothelial growth factor
IVT: Intravitreal injection
VRI: Vitreal-retina interface
RPE-TJ: Retinal pigment epithelium tight junction
TRG: Temperature-responsive hydrogel
HA: Hyaluronic acid
RB-M: Rhein and baicalein-coloaded microemulsion
Bor/RB-M: Borneol-decorated rhein and baicalein-coloaded microemulsion
RB-M@TRG: Rhein and baicalein-coloaded microemulsion-doped intravitreal-injectable hydrogel depot
Bor/RB-M@TRG: Borneol-decorated rhein and baicalein-coloaded microemulsion-doped intravitreal-injectable hydrogel depot
Sd III-M: Sudan III-labeled microemulsion
Sd III-M @TRG: Sd III-labeled microemulsion-doped intravitreal-injectable hydrogel depot
ARPE-19: Human retinal pigment epithelial
HUVECs: Human umbilical vein endothelial cells
ELISA: Enzyme linked immunosorbent assay
HRMS: High resolution mass spectrometry
^1^H-NMR: Hydrogen spectrum nuclear magnetic resonance
FT-IR: Fourier transform infrared spectroscopy
MWCO: Molecular weight cut-off
DLS: Dynamic light scattering
TEM: Transmission electron microscopy
C6: Coumarin 6
C6-M: C6-loaded microemulsion
Bor/C6-M: Borneol-decorated C6-loaded microemulsion
Bor/C6-M@TRG: Borneol-decorated C6-loaded microemulsion-doped intravitreal-injectable hydrogel depot
PBS: Phosphate buffer solution
HE: Hematoxylin and eosin
TUNEL: TdT-mediated dUTP nick end labeling
TBHP: Tert-Butyl hydroperoxide
SA: Succinic anhydride
Bor: Borneol
Bor-PEG400-LA: Borneol-conjugated polyethylene glycol laurate
SA-Bor: Butanediyl borneol
DMAP: 4-Dimethylaminopyridine
DCC: Dicyclohexylcarbodiimide
HPLC: High-performance liquid chromatography
G': Storage moduli
G″: Loss moduli
SEM: Scanning electron microscopy
SV: Simulated vitreous
CLSM: Confocal laser scanning microscope
MDA: Malondialdehyde
SOD: Superoxidedismutase
TNF-α: Tumor necrosis factor-α
IL-1β: Interleukin-1β
OCT: Optical coherence tomography
FFA: Fundus fluorescence angiography
RBC: Red blood cells
