# Supplementary material for: An intravitreal-injectable hydrogel depot doped borneol-decorated dual-drug-coloaded microemulsions for long-lasting retina delivery and synergistic therapy of wAMD
Source: J Nanobiotechnology. 2023 Mar 1;21:71. doi: 10.1186/s12951-023-01829-y (PMC9976542; doi:10.1186/s12951-023-01829-y)
Supplement: Supplementary file 1 — Additional file 1: Figure S1. FT-IR analysis of Bor-PEG400-LA. Figure S2. Particle size and PDI of Blank-M with different surfactant. All the quantification data are shown as mean ± SD, n = 3. ***P < 0.001 vs (RH40) Blank-M. Figure S3. Size distribution of Bor/M studied DLS. The left and right inserted pictures are Blank-M solution and its TEM image. The bar is 100 nm. Figure S4. Size distribution of RB-M studied DLS. The left and right inserted pictures are RB-M solution and its TEM image. The bar is 100 nm. Figure S5. Stability of Sd III-M@TRG in simulated vitreous. (A–E) Feedings of HA (800–1500 kDa) were 0.4%, 0.6%, 0.8%, 1.0%, and 1.2%, respectively. (F–J) Feedings of HA (1500–2500 kDa) were 0.4%, 0.6%, 0.8%, 1.0%, and 1.2%, respectively. Figure S6. Rheological tests of Blank TRG. (A) Variation curves of G″ and G' with temperature; (B) Variation curve of compound viscosity coefficient and temperature. Figure S7. Cell apoptosis of H2O2-induced RPE after treatments with different formulations. All the quantification data are shown as mean ± SD, n = 6. **P < 0.01 vs Model. Figure S8. Migration of HUVECs studied by Transwell. All the quantification data are shown as mean ± SD, n = 6. **P < 0.01 vs Control; #P < 0.05. Figure S9. Invasion of HUVECs studied by Transwell. Data are shown as mean ± SD, n = 6. *P < 0.05, **P < 0.01 vs Control; ##P < 0.01. Figure S10. FFA images of wAMD model mice treated with RH and BCL at day 14. IB4 staining of the RPE-choroid complex layer. The part circled in red is the CNV area, and the part circled in white is the optic disc. The bar is 200 μm. Figure S11. HE staining of eyeballs of healthy SD rats treated with different formulations at day 21. The bar is 200 μm. Figure S12. Hemolysis tests of Bor/RB-M. Data are shown as mean ± SD, n = 3. [file 12951_2023_1829_MOESM1_ESM.docx]

**Additional Information**

**An** **intravitreal-injectable hydrogel depot doped borneol-decorated dual-drug-coloaded microemulsions for long-lasting retina delivery and synergistic therapy of wAMD**

Wenting Su^1#^, Congyan Liu^1,2#^, Xi Jiang^1^, Yanli Lv^1,2^, Qin Chen^3^, Jiachen Shi^1^, Huangqin Zhang^1,2^, Qiuling Ma^1^, Chang Ge^1^, Fei Kong^1^, Xiaoqi Li^1,2^, Yuping Liu^1,2^, Yan Chen ^1,2^, Ding Qu^1,2,*^

^1^Affiliated Hospital of Integrated Traditional Chinese and Western Medicine, Nanjing University of Chinese Medicine, Nanjing, 210028, China.

^2^Jiangsu Province Academy of Traditional Chinese Medicine, Nanjing, 210028, China.

^3^Department of Ophthalmology, Nanjing Drum Tower Hospital, the Affiliated Hospital of Nanjing University Medical School, Nanjing, 210008, China.

*Corresponding author: Ding Qu, Ph.D., Professor, Jiangsu Province Academy of Chinese Medicine, 100 Shizi Road, Nanjing 210028, China. E-mail: quding1985@hotmail.com;

*Correspondence to: Ding Qu (E-mail: quding1985@hotmail.com);

^#^These authors contributed equally to this work.


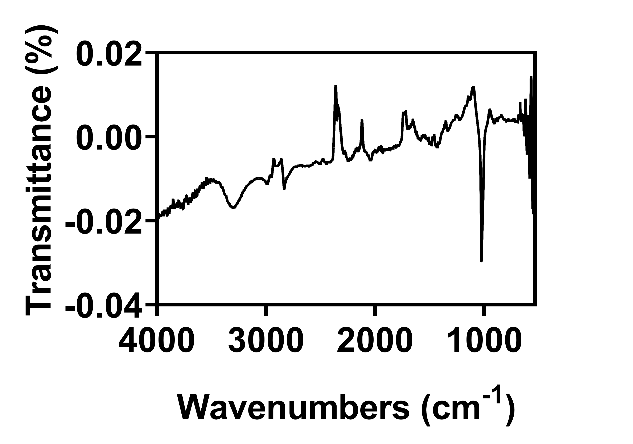


**Figure S1.** FT-IR analysis of Bor-PEG_400_-LA.


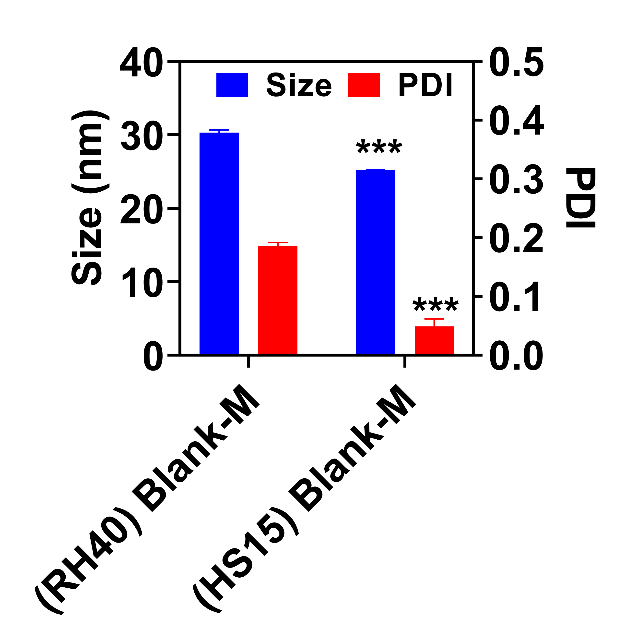


**Figure S2.** Particle size and PDI of Blank-M with different surfactant. All the quantification data are shown as mean ± SD, n = 3. ****P* < 0.001 vs (RH40) Blank-M.


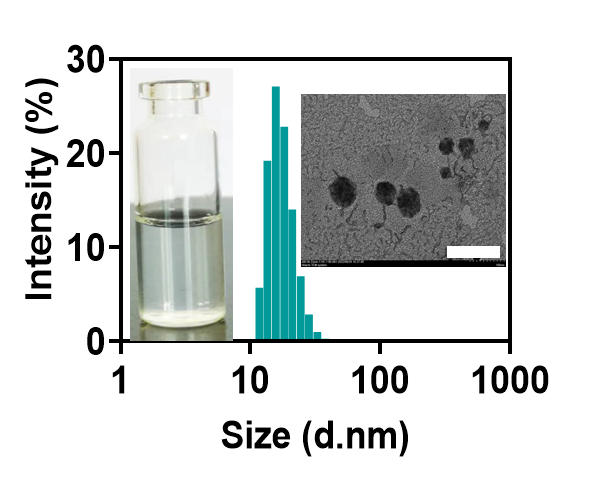


**Figure S3.** Size distribution of Bor/M studied DLS. The left and right inserted pictures are Blank-M solution and its TEM image. The bar is 100 nm.


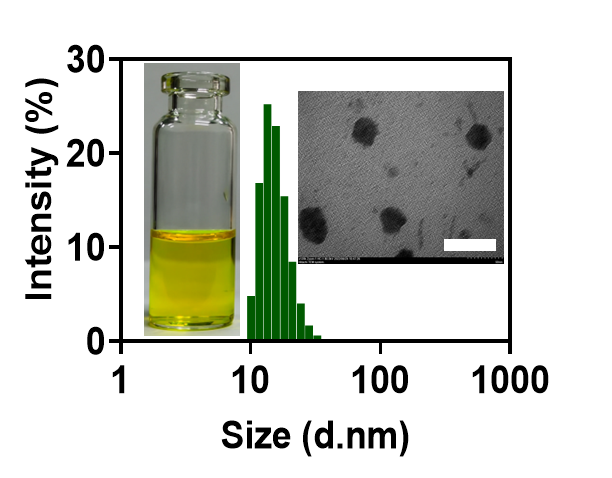


**Figure S4.** Size distribution of RB-M studied DLS. The left and right inserted pictures are RB-M solution and its TEM image. The bar is 100 nm.


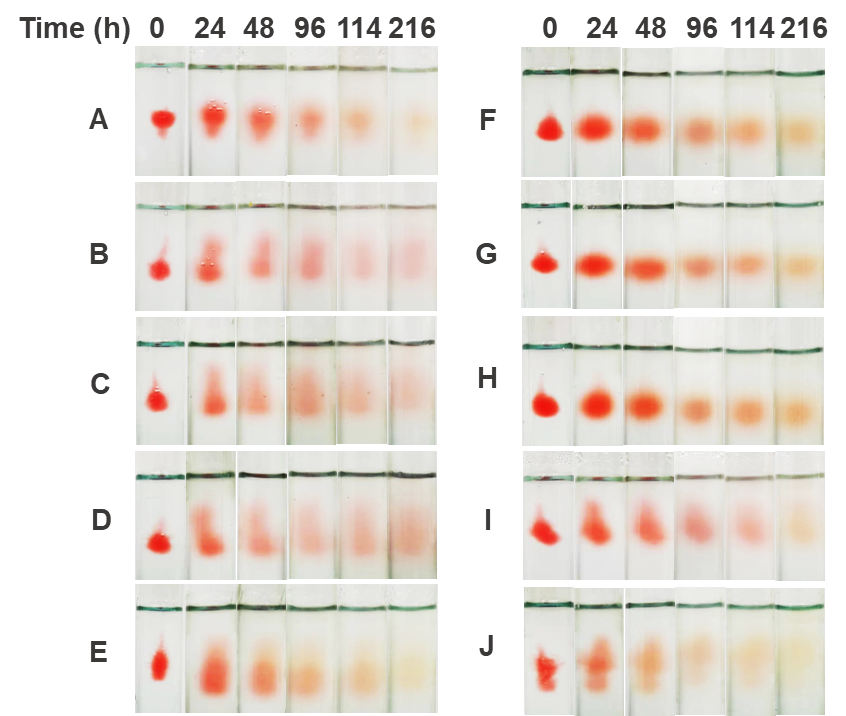


**Figure S5.** Stability of Sd III-M@TRG in the SV. (A-E) Feedings of HA (800 ~ 1500 kDa) were 0.4%, 0.6%, 0.8%, 1.0%, and 1.2%, respectively. (F-J) Feedings of HA (1500 ~ 2500 kDa) were 0.4%, 0.6%, 0.8%, 1.0%, and 1.2%, respectively.


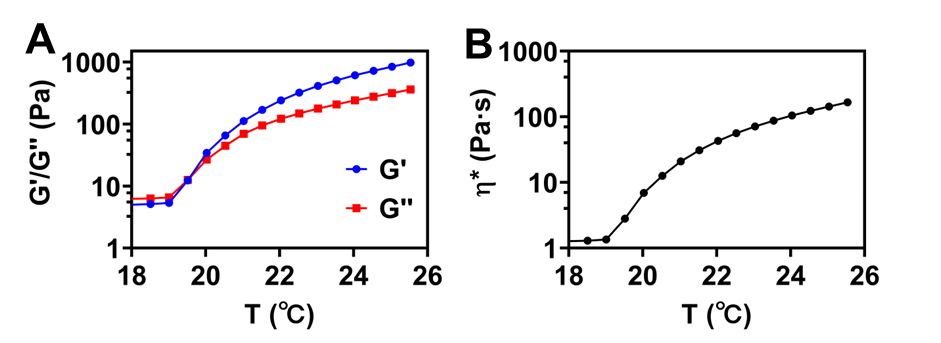
**Figure S6.** Rheological tests of blank TRG. (A) Variation curves of G″ and G′ with temperature. (B) Variation curve of compound viscosity coefficient and temperature.


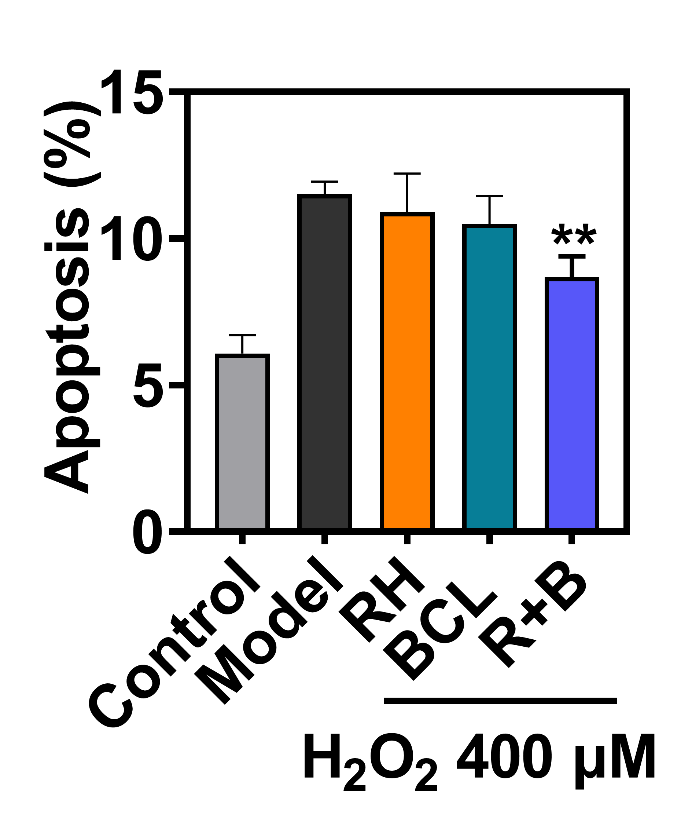


**Figure S7.** Cell apoptosis of H_2_O_2_-induced RPE after treatments with different formulations. All the quantification data are shown as mean ± SD, n = 6. ***P* < 0.01 vs Model.


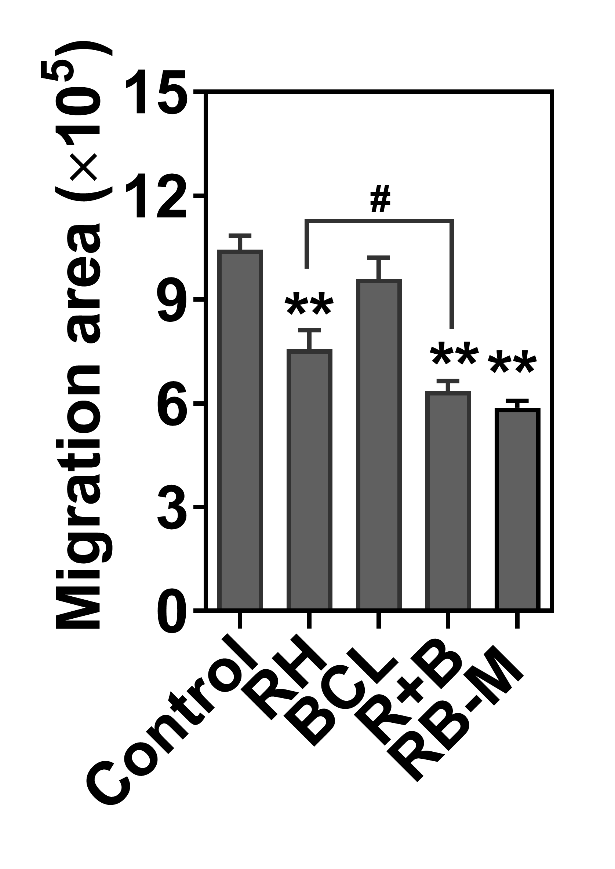


**Figure S8.** Migration of HUVECs studied by Transwell. All the quantification data are shown as mean ± SD, n = 6. ***P* < 0.01 vs Control; ^#^*P* < 0.05.


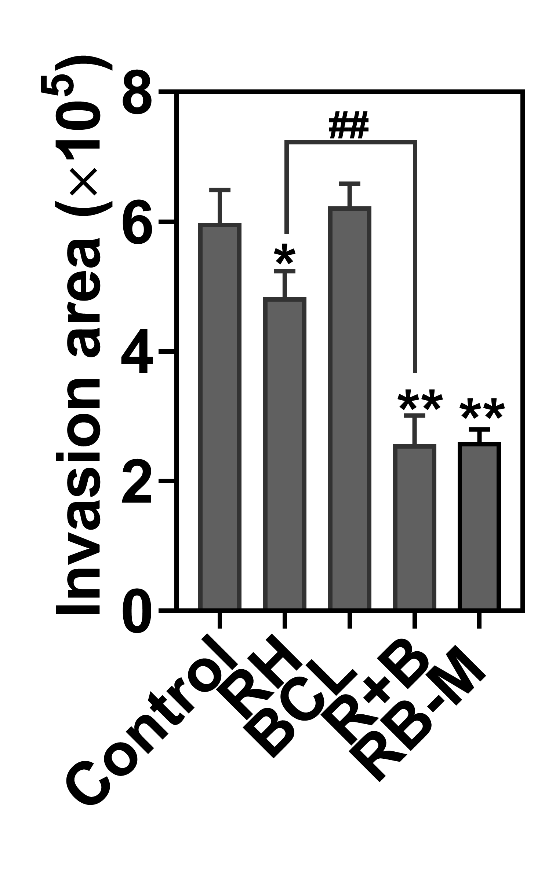


**Figure S9.** Invasion of HUVECs studied by Transwell. Data are shown as mean ± SD, n = 6. **P* < 0.05, ***P* < 0.01 vs Control; ^##^*P* < 0.01.


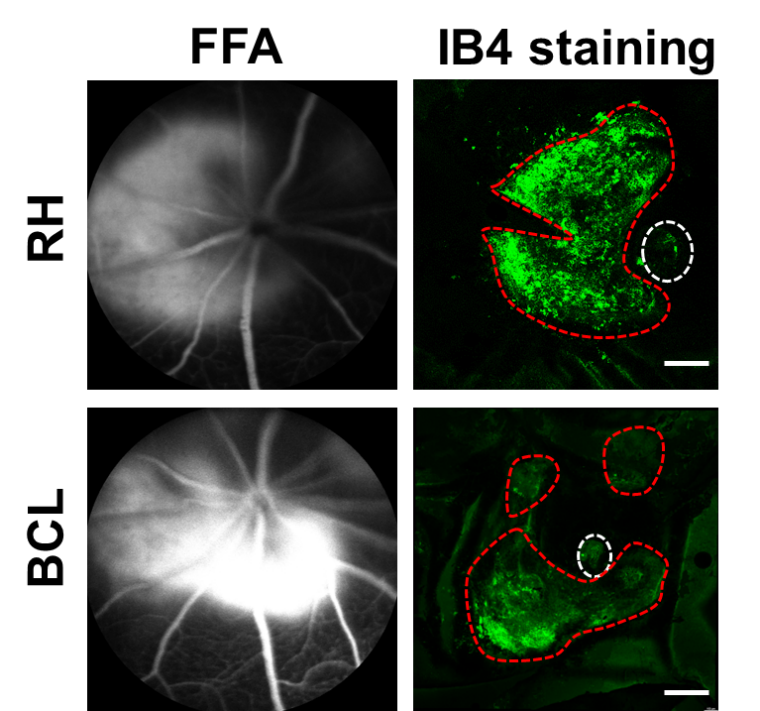


**Figure S10.** FFA images of wAMD model mice treated with RH and BCL at day 14. IB4 staining of the RPE-choroid complex layer. The part circled with red is the CNV area, and the part circled with white is the optic disc. The bar is 200 μm.


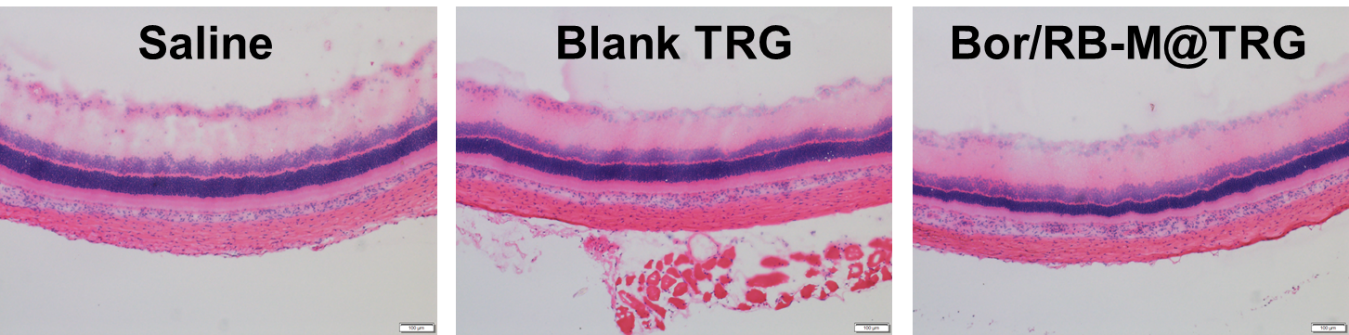


**Figure S11.** HE staining of eyeballs of healthy SD rats treated with different formulations at day 21. The bar is 100 μm.


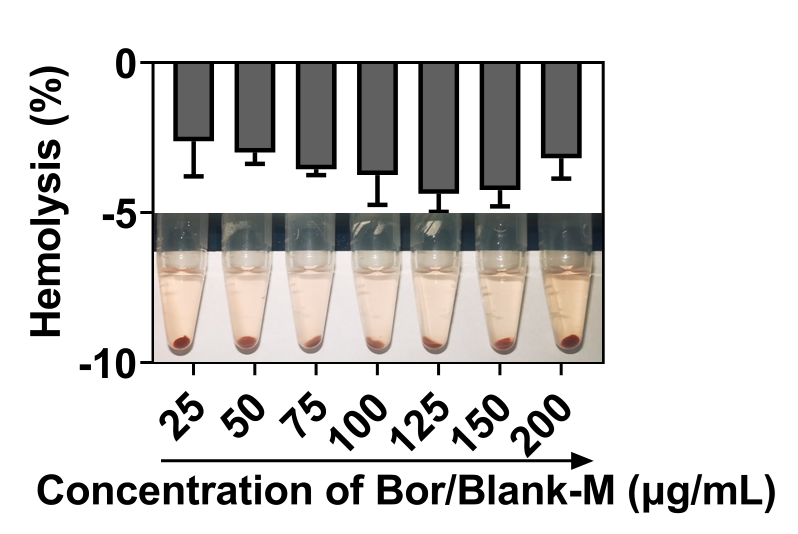


**Figure S12.** Hemolysis tests of Bor/RB-M. Data are shown as mean ± SD, n = 3.
